# Comparisons of cell proliferation and cell death from tornaria larva to juvenile worm in the hemichordate *Schizocardium californicum*

**DOI:** 10.1186/s13227-022-00198-1

**Published:** 2022-06-06

**Authors:** Paul Bump, Margarita Khariton, Clover Stubbert, Nicole E. Moyen, Jia Yan, Bo Wang, Christopher J. Lowe

**Affiliations:** 1 Hopkins Marine Station, Department of Biology, Stanford University, Pacific Grove, CA USA; 2 Department of Bioengineering, Stanford University, Stanford, CA USA; 3 California State University of Monterey Bay, Monterey, CA USA; 4 CZ Biohub, San Francisco, CA USA

**Keywords:** Metamorphosis, Hemichordate, Tornaria, Cell proliferation, Cell death

## Abstract

**Background:**

There are a wide range of developmental strategies in animal phyla, but most insights into adult body plan formation come from direct-developing species. For indirect-developing species, there are distinct larval and adult body plans that are linked together by metamorphosis. Some outstanding questions in the development of indirect-developing organisms include the extent to which larval tissue undergoes cell death during the process of metamorphosis and when and where the tissue that will give rise to the adult originates. How do the processes of cell division and cell death redesign the body plans of indirect developers? In this study, we present patterns of cell proliferation and cell death during larval body plan development, metamorphosis, and adult body plan formation, in the hemichordate *Schizocardium californium* (Cameron and Perez in Zootaxa 3569:79–88, 2012) to answer these questions.

**Results:**

We identified distinct patterns of cell proliferation between larval and adult body plan formation of *S. californicum*. We found that some adult tissues proliferate during the late larval phase prior to the start of overt metamorphosis. In addition, using an irradiation and transcriptomic approach, we describe a genetic signature of proliferative cells that is shared across the life history states, as well as markers that are unique to larval or juvenile states. Finally, we observed that cell death is minimal in larval stages but begins with the onset of metamorphosis.

**Conclusions:**

Cell proliferation during the development of *S. californicum* has distinct patterns in the formation of larval and adult body plans. However, cell death is very limited in larvae and begins during the onset of metamorphosis and into early juvenile development in specific domains. The populations of cells that proliferated and gave rise to the larvae and juveniles have a genetic signature that suggested a heterogeneous pool of proliferative progenitors, rather than a set-aside population of pluripotent cells. Taken together, we propose that the gradual morphological transformation of *S. californicum* is mirrored at the cellular level and may be more representative of the development strategies that characterize metamorphosis in many metazoan animals.

**Supplementary Information:**

The online version contains supplementary material available at 10.1186/s13227-022-00198-1.

## Background

The development of animal body plans has largely been informed by research in a few key model species that pattern the adult body plan during embryogenesis, a strategy termed direct development. However, this type of development is not representative of many animal groups, where embryogenesis gives rise to a larva with a body plan distinct from that of the adult, a strategy called indirect development [2–4]. During direct development, the adult is formed directly from the embryo, while in indirect development, embryonic processes give rise to a larval body plan that later transforms into the adult. This transformation between larvae and adults is a developmental process known as metamorphosis, which is characterized by the loss of larval-specific structures and the emergence of adult structures [5–11]. The prevalence of this developmental strategy across animal phyla clearly demonstrates that a better mechanistic understanding of indirect development is critical for a more complete understanding of the developmental basis of body plan evolution.

Many marine organisms utilize indirect development, developing first as larvae that feed and grow before reaching metamorphosis [12, 13]. In species, such as gastropods with veliger larvae, the morphological difference between larval and adult body plans is not very pronounced, because in these organisms, metamorphosis represents a major shift in ecological niche but not a large morphological change [14, 15]. At the other end of the spectrum, as is found in some echinoids, larval and adult body plans can be radically different in organization with a “catastrophic metamorphosis.” In this case, the adult develops as a rudiment within the larva and metamorphosis results in a complete reorganization of the body around new developmental axes in addition to the loss of larval structures [5, 16, 17]. Similarly, in some nemertean worms, with a pilidium larva, the adult develops from several rudiments, and metamorphosis culminates with the juvenile consuming the larval tissues [17, 18]. However, metamorphosis in species with distinct larval and adult body plans does not always involve a segregated rudiment or cataclysmic metamorphosis — instead larval tissue seems to be remodeled rapidly into the adult without obvious drastic histolysis of the larval body plan [2, 15, 19]. In this type of metamorphosis, do adult structures originate from a small population of proliferative cells? What is the fate of larval tissues? Indirect-developing hemichordates represent this particular type of metamorphosis and provide an opportunity to explore this type of developmental strategy.

Hemichordates are composed of two classes, the solitary enteropneust worms and the largely colonial, tube-dwelling pterobranchs [20–23]. While the position of hemichordates as sister to the echinoderms and closely related to chordates has been well established [24–27], new studies have challenged this position [28]. Within the enteropneusts, one family, the Harrimaniiidae, are direct developers, while the families Spengelidae and Ptychoderidae, are indirect developers with a distinct larval body plan called the tornaria. Morphological studies of tornaria larvae and their counterpart adult bodies can be traced back to the late 1800s [29, 30] and more recent morphological descriptions of larval and adult body plans have been carried out in a range of enteropneust species; *Ptychodera flava* [31, 32], *Balanoglossus misakiensis* [33], *Balanoglossus simodensis* [34] and *S. californicum* [35]. In these species, the tornaria larva is formed following embryogenesis, while the benthic adult body plan forms by metamorphosis following an extended planktonic period [31, 33–36]. Studies of hemichordate complex life cycles have largely been based on morphological characters, with some descriptive patterning studies [37–53]*.* However, the cellular and developmental mechanisms through metamorphosis remain largely uncharacterized. For example, we do not know whether the adult is formed by transformation of larval tissues via transdifferentation or by proliferation of adult-specific cells following large-scale larval cell death.

To begin to address these questions, we characterized proliferation and cell death through the development and metamorphosis of *S. californicum*. For the purpose of our study, we define metamorphosis in *S. californicum* as an overt morphological event. In a rapid 48-h period a swimming planktonic larva transforms into a burrowing benthic adult as larval ectoderm compacts and condenses over the underlying mesoderm and endoderm[35]. We found distinct patterns of cell proliferation between larval and adult body plans and that the start of a clear overt metamorphosis corresponded with an increase in cell death. To then determine if there were distinct genetic markers of proliferative cells, and if those markers differed between life history stages, we deployed an irradiation strategy to deplete proliferative cells and found a number of differentially expressed transcripts.

## Results

### Patterns of proliferation in larval and adult body plans.

**Fig. 1:**
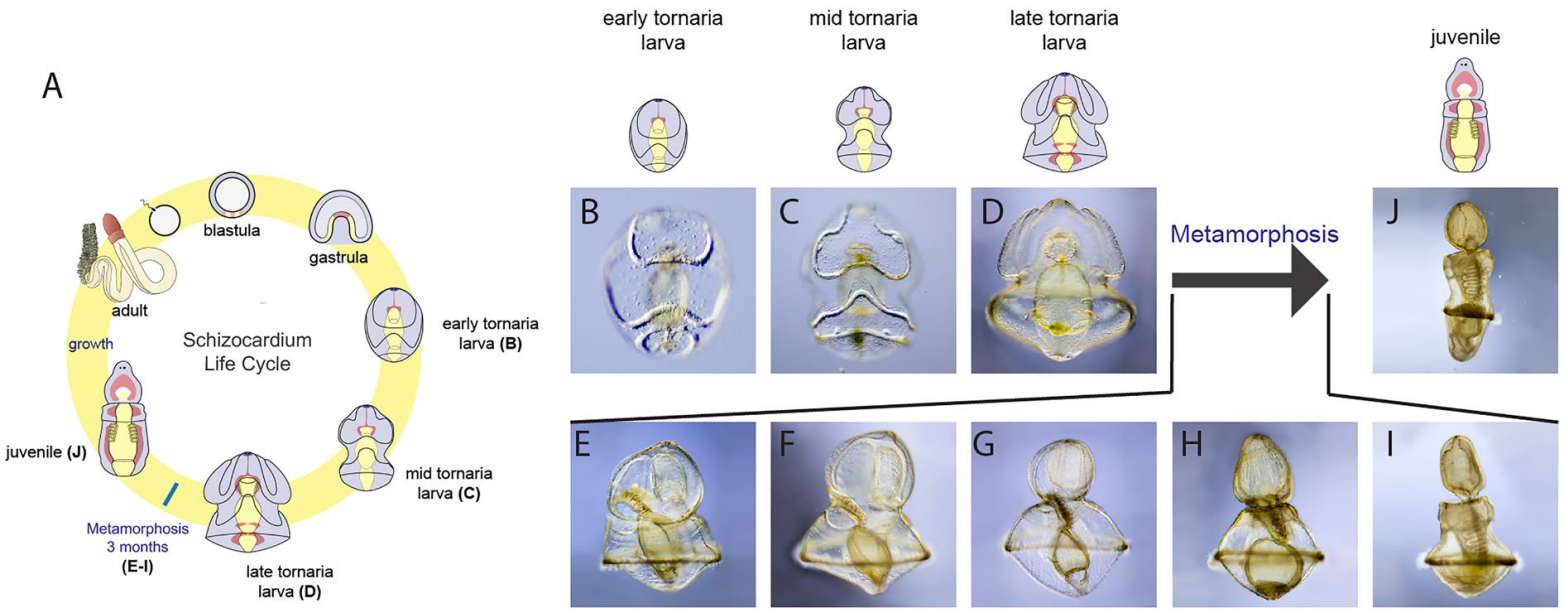
Larval development and metamorphosis of *S. californicum.* **A** Schematic of the complex life cycle of the indirect developing *S. californicum* (modified from Ref. [35]. **B**–**J** Light microscopy of the complex life cycle of the indirect developing *S. californicum* (from [35]). **B** early tornaria larva, **C** mid tornaria larva, **D** late tornaria larva, **E**–**I** process of metamorphosis, **J** juvenile

We wanted to test whether patterns of cellular proliferation involved in the development of the planktonic larva were similar or different to those during the development of the benthic juvenile. To describe the distribution of proliferative cells throughout the development of *S. californicum,* we assessed the incorporation of the thymidine analog 5-ethynyl-29-deoxyuridine (EdU), which labels cells in S phase [54], during a range of developmental stages: early larval development (Fig. 1B), mid larval development (Fig. 1C), late larval development (Fig. 1D), metamorphosis (Fig. 1E–I), and in juvenile development (Fig. 1J).

**Fig. 2:**
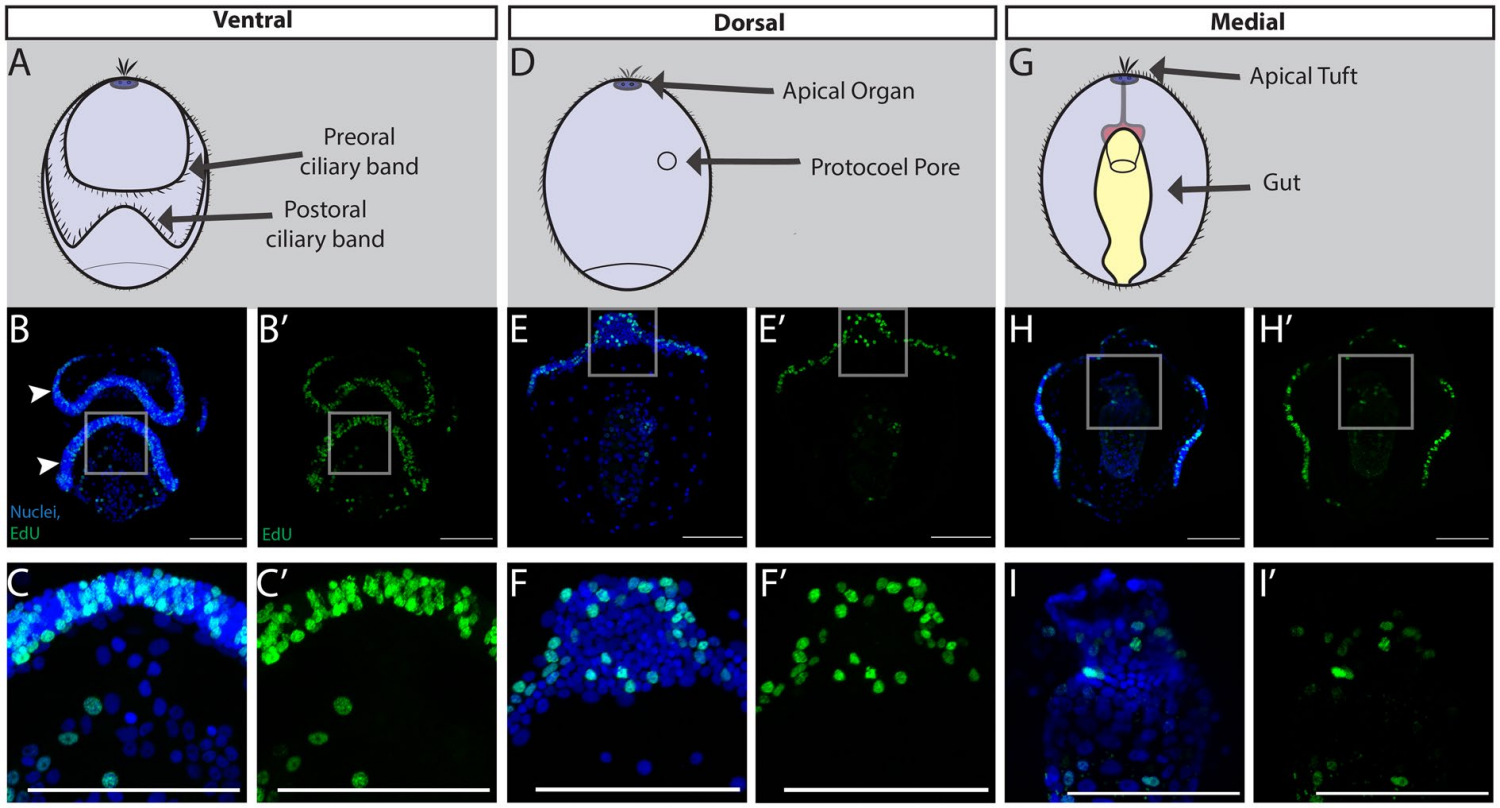
Cell proliferation throughout early larval development of *S. californicum*. All: anterior up; blue = Hoechst, green = EdU; scale bar is 100um. Schematics of ventral (**A**), dorsal (**D**), and medial (**E**) views with key structures labeled. EdU staining with maximum intensity projections showing ventral (**B**), dorsal (**E**), and medial **H** sections. **C**–**I** insets. White arrowheads denote preoral ciliary band and postoral ciliary band

The earliest larval developmental stage consists of a tightly packed ciliary band that loops around the larva, a thin wide squamous epithelium, an apical tuft, and a tripartite gut (Figs. 1B, 2). On the ventral surface we detected EdU^+^ cells throughout the preoral and postoral loops of the circumoral ciliary band (Fig. 2B, C). The ciliary band is used for both swimming and particle capture at this stage [55–57] and makes up a large percentage of the ectoderm. We tested whether the ciliary bands were more proliferative than the general ectoderm, or simply had higher cell densities. The ciliary bands were densely packed with nuclei: there are ~ 59% greater number of cells in the ciliary bands versus all other tissues (paired *t* test, p = 0.016) and they were also more proliferative with ~ 22% more Edu^+^ cells than all other tissues (paired *t* test, p = 0.008) (Additional file 1: S1A, B). This suggests that while the ciliary bands are nuclei-dense regions, they appear to be some of those most proliferative structures at this stage. This pattern aligns with what has been observed in the ciliary bands of other Ambulacrarians, such as the bipinnaria larvae of *Pisaster ochraceus* and *Patiria miniata* [58]*.* On the dorsal side of the larva in the most anterior regions, EdU^+^ cells were detected around the apical organ (Fig. 2E, F), a prominent structure of the larval nervous system [59–62]. Other important proliferative structures of larvae include the digestive tract, where microalgae that have been captured by the ciliary bands pass from the mouth into the pharynx, and finally into the stomach, where they are digested (Fig. 2H, I). In general, at this early larval stage most regions and tissues contain proliferative cells.

**Fig. 3:**
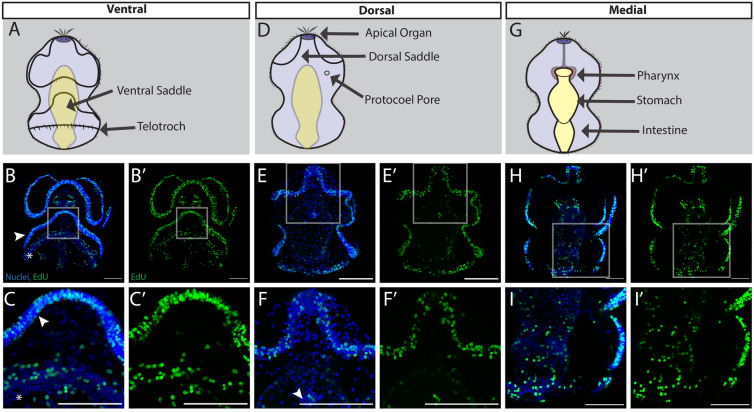
Cell proliferation throughout mid larval development of *S. californicum*. All: anterior up; blue = Hoechst, green = EdU; scale bar is 100um. Schematics of ventral (**A**), dorsal (**D**), and medial (**E**) views with key structures labeled. EdU staining with maximum intensity projection showing ventral (**B**), dorsal (**E**), and medial (**H**) sections. **C**–**I** insets. White arrowhead in (**B** and **C**) denote postoral ciliary band, white asterisk denotes telotroch. White arrowhead in (**F**) denotes protocoel pore

As the tornaria continues to grow and reaches the middle of larval development (Figs. 1C, 3), defined by the differentiation of dorsal and ventral saddles as well as the emergence of the posterior telotroch, proliferation continued throughout the ciliary bands. This was most apparent ventrally in the preoral and post-oral ciliary bands (Fig. 3B). Proliferative cells were detected in the developing telotroch, the posterior locomotory ciliary band (Fig. 3C). The telotroch is one of the most distinctive structures of the hemichordate tornaria with long compound cilia that beat to propel the larva through the water [57]. On the dorsal surface, the protocoel pore was proliferative at this stage (Fig. 3E, F). This structure is a portion of the larval protonephridial system, an excretory system that uses a cilia-driven flow for ultrafiltration of coelomic fluid from the protocoel [63, 64]. Finally, at this stage the last notable structure is the tripartite gut, composed of pharynx, stomach and intestine, which continued to proliferate and grow (Fig. 3H, I).

**Fig. 4:**
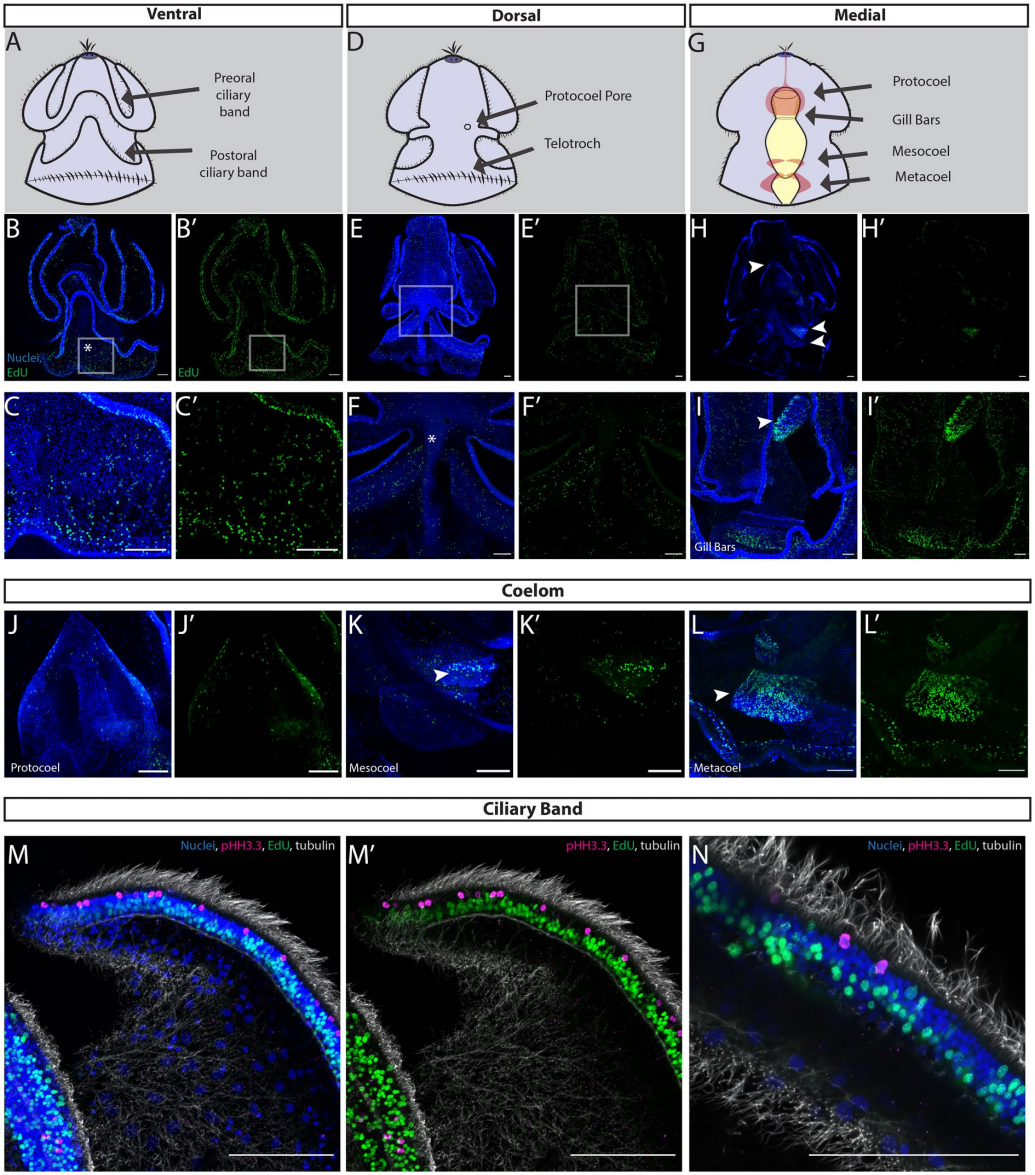
Cell proliferation in late larval development of *S. californicum*. All: anterior up; blue = Hoechst, green = EdU; scale bar is 100 um. Schematics of ventral (**A**), dorsal (**D**), and medial (**G**) views with key structures labeled. EdU staining with maximum intensity projection ventral (**B**), dorsal (**E**), and medial (**H**) sections. **B** late larva ventral surface, **C** inset of (**B**), highlights ventral posterior epidermis and ciliary band **E** late larva dorsal surface, **F** inset of (**E**) highlights dorsal cord, marked by white asterisk, **H** late larva medial section, arrowheads highlight regions that will give rise to protocoel, mesocoel, and metacoel. **I** inset of lateral view of late larva medial section, arrowhead highlights gill bars. **J** protocoel, mesoderm that will form the proboscis, **K** mesocoel, mesoderm that will form the collar, **L** metacoel, mesoderm that will form the trunk. **M**, **N** distribution of anti-histone H3 (phospho S10) and EdU positive cells in ciliary bands, magenta = pHH3.3, grey = acetylated tubulin

Close to metamorphosis, the tornaria larva reach full size (~ 3 mm) and form two additional coelom pairs, the mesocoels and metacoels, and the precursors to the gill slits [65, 66] (Figs. 1D, 4), and we observed a notable shift in proliferative patterns from earlier developmental stages. Proliferative cells were still distributed throughout the ventral ectoderm, both in the ciliary bands, but now also more broadly in the squamous epithelium between the ciliary bands (Fig. 4B). There were also a number of EdU^+^ cells distributed broadly throughout the posterior ectoderm of the larva, which is a territory that will compact and elongate during metamorphosis (Fig. 4C). Across the dorsal surface of the late larva, there were numerous proliferative cells distributed throughout the epithelium (Fig. 4E). There were EdU^+^ cells throughout the telotroch and on either side of the dorsal midline, where the dorsal cord was beginning to form (Fig. 4F). Perhaps most interestingly, at this stage, the gut stopped proliferating and EdU^+^ cells were detected within the forming adult structures (Fig. 4H). In particular, we observed EdU^+^ cells in the anlage of the gill slits, which are a prominent endomesoderm feature of the juvenile body plan that are not yet functional in the late larva [67] (Fig. 4I). EdU^+^ cells were also enriched in the single anterior protocoel (Fig. 4J), and more posterior paired mesocoels (Fig. 4K) and metacoels (Fig. 4L), which will later form the adult mesodermal derivatives of the proboscis, collar, and trunk, respectively. In line with previous morphological observations, in late larvae, structures of the juvenile body plan began to proliferate to build the adult anatomical structures ahead of metamorphosis [35].

We also looked in more detail at the proliferative patterns in the ciliary band (Fig. 4M, N). To achieve this, we coupled our EdU detection with immunofluorescence staining of acetylated tubulin to visualize cilia and phosphorylated serine 10 of histone H3 (pHH3.3), which marks cells in G2/M phase. We found that proliferative cells display distinct spatial distribution with a row of EdU^+^ cells at the base, then a row of differentiating phosophohistone h3.3 cells that are lateral to the cilia (Fig. 4M, N). This regional localization of EdU^+^ cells in relationship to the differentiating phosophohistone h3.3 cells suggested that there could be a specific population of proliferative cells that give rise to the ciliary bands.

### Proliferative patterns shift at metamorphosis

**Fig. 5:**
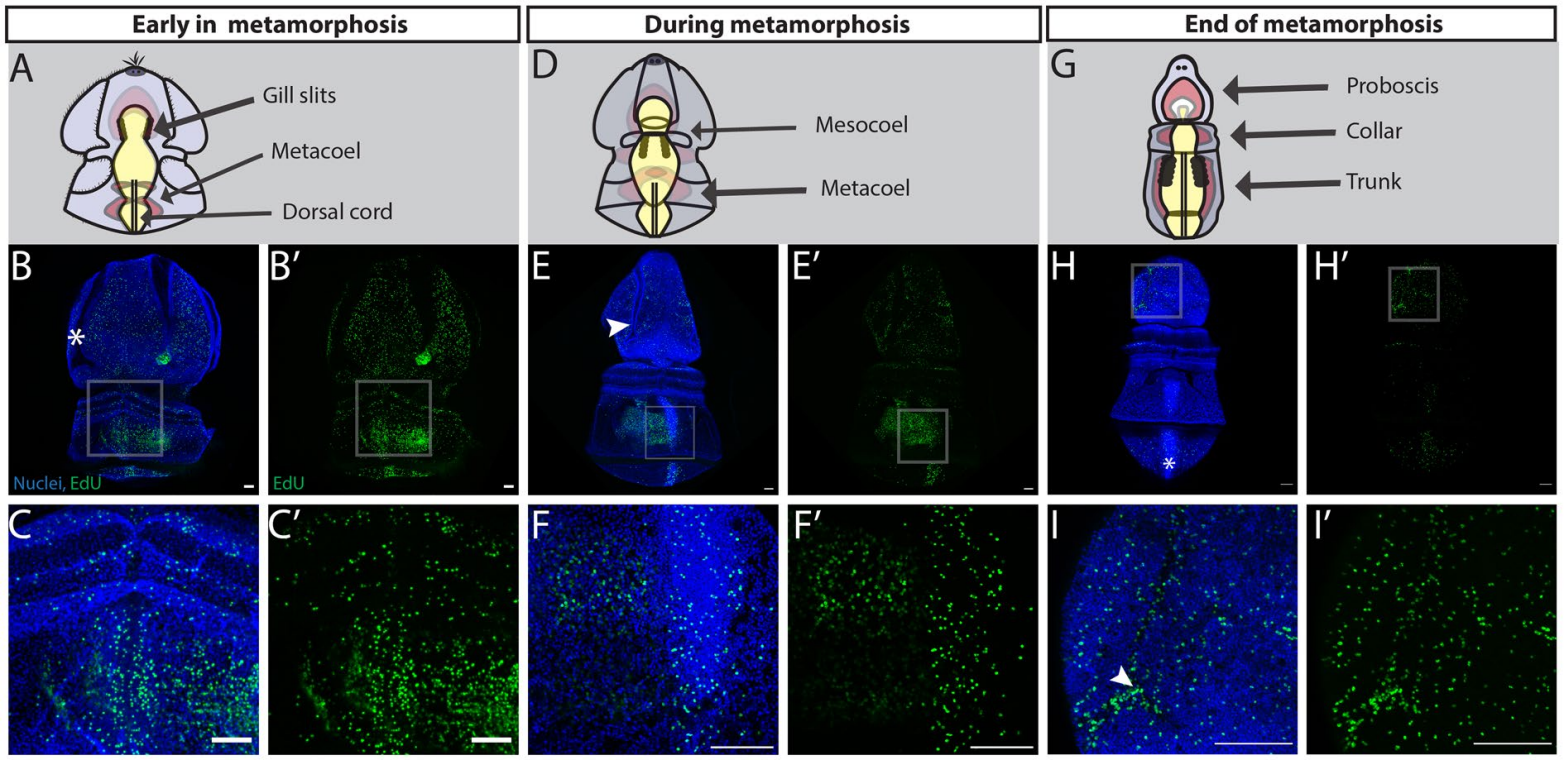
Cell proliferation throughout the metamorphosis of *S. californicum*. All: anterior up; dorsal view; blue = Hoechst, green = Edu; scale bar is 100um. Schematics of ventral (**A**), dorsal (**D**), and medial (**G**) views with key structures labeled. EdU staining with maximum intensity projection: **B** Early in metamorphosis, white asterisk marks lateral food groove **C** Inset of (**B**), EdU positive cells are distributed around and in the dorsal cord. **E** Middle of metamorphosis, white arrowhead marks lateral food groove. **F** Inset of (**E**), EdU positive cells are found in the dorsal cord and mesocoel. **H** End of metamorphosis, asterisk marks dorsal cord. **I** Inset of (**H**), white arrowhead highlights EdU positive cells are distributed throughout the lateral grooves

The first morphological indication of the onset of metamorphosis in *S. californicum* is the compaction and reorganization of the larval epidermis and an expansion of all the coeloms, which results in a decrease of the blastocoelar space (Fig. 1E, F) [35]. Early in metamorphosis, the ectoderm of the primary ventral lobe and primary dorsal lobe compact around the lateral food groove, as has been observed in *P. flava* [66], and EdU^+^ cells were distributed throughout several regions of the ectoderm (Fig. 5B). EdU^+^ cells were detected in the postoral field and primary dorsal saddle that give rise to both the proboscis and around the thickening collar (Fig. 5B). At this stage, EdU^+^ cells were also found in the collar and posterodorsally in the region of the developing dorsal cord (Fig. 5C). On the ventral surface, EdU^+^ cells showed a similar distribution to the dorsal side with proliferative cells in the preoral field, around the collar, in the anlage of the gill slits and in the epidermis, where the ventral cord eventually forms (Additional file 1: S1C).

Metamorphosis then proceeded with the prospective proboscis ectoderm continuing to thicken as the blastocoel was reduced, bringing it in contact with the expanding anterior coelom (Fig. 1G, H). The posterior ectoderm continued to expand as the forming trunk continued to elongate. At this stage, ectodermal proliferation continued in the general epidermis of the proboscis but was absent from the remnants of the ciliary bands (Fig. 5E). The epidermis of the proboscis transformed into a columnar organization as the larva began to take on a more vermiform shape. Other proliferative regions at this stage included the developing gill slits, the metacoels, and the dorsal cord (Fig. 5F). At this stage, proliferation on the ventral surface occurred in the anterior ectoderm, similar to the dorsal surface, absent from where the ciliary bands had been (Additional file 1: S1D). EdU^+^ cells were also detected around the collar and around the field, where the ventral cord forms (Additional file 1: S1D).

Finally, metamorphosis concluded as the blastocoelar space of the proboscis was eliminated bringing the mesoderm and ectoderm in direct contact, the ectoderm of the proboscis and collar transformed into a columnar epithelium, and the posterior coeloms expanded and differentiated as the trunk was elongating and narrowing (Fig. 1I). At this stage, we detected proliferative cells specifically in the proboscis, the collar, dorsal cord, and more broadly below the telotroch in the most posterior ectoderm and mesoderm (Fig. 5H). In the proboscis ectoderm there were EdU^+^ cells distributed throughout as well as a clear enrichment of EdU^+^ cells in the lateral groove, the region that had previously been the larval food groove (Fig. 5I). A lateral view of this stage at metamorphosis, highlighted cell proliferation in the gill slits and gut as well as the dorsal and ventral midlines that give rise to the nerve cords (Additional file 1: S1E).

**Fig. 6:**
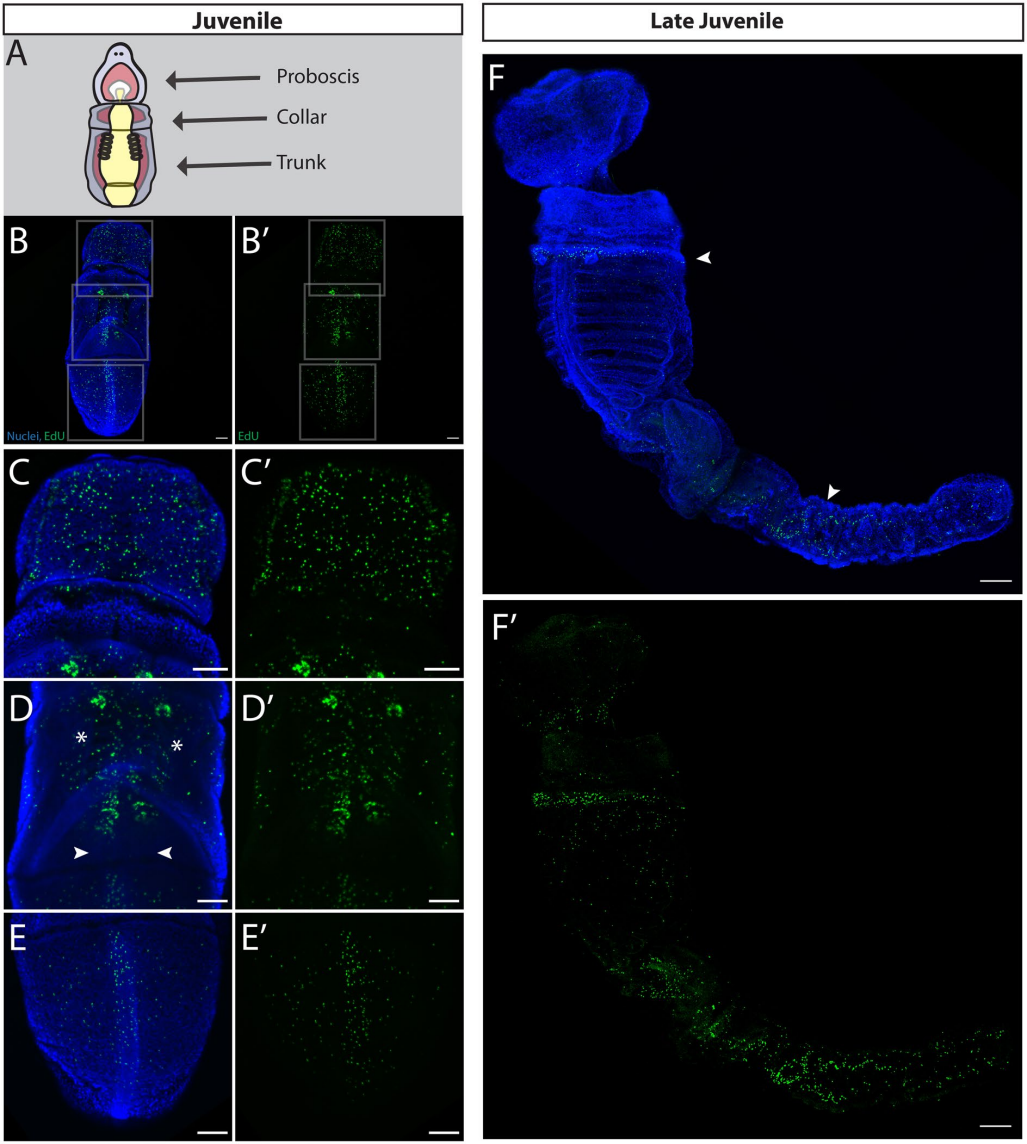
Cell proliferation in juveniles of *S. californicum*. All: anterior up; dorsal view; blue = Hoechst, green = Edu; scale bar is 100 um. Schematic of juvenile (**A**) with key structures labeled. EdU staining with maximum intensity projection: **B** End of metamorphosis. **C** Highlights regions of (**B**), EdU positive cells are distributed throughout the proboscis. **D** Highlights regions of (**B**), EdU positive cells are distributed throughout the gill bars. **E** Highlights regions of (**B**), EdU positive cells are distributed throughout the dorsal cord. **F** Continuing development of the juvenile body plan. Arrowheads mark the base of the collar and expanding trunk

In three main regions of the newly formed juvenile (Fig. 1J), cell proliferation was detected in the proboscis, collar, gill pores, gill bars, and trunk (Fig. 6B). In the anterior of the juvenile, EdU^+^ cells were localized to the epidermis and line the lateral groove and anterior collar (Fig. 6C). This region of the animal is highly innervated [35]. Proliferative cells were also found in the dorsal gill pores which have perforated to allow water flow through the gill slits and out the gill pores (Fig. 6D). Finally in the posterior of the newly formed juvenile, proliferative cells were located along the dorsal cord of the trunk (Fig. 6E). At this stage on the ventral surface, we detected EdU^+^ cells in the proboscis, the gill slits, and in the ventral cord (Additional file 1: S1F). To see if these patterns of juvenile growth continued well after metamorphosis, we grew animals in sand for several weeks and repeated the EdU labeling, clearing the tissue to make it possible to visualize the distribution of proliferation in larger, thicker tissue. In continued juvenile growth (Fig. 6F) proliferative cells were found at the base of the collar coincident with serotonergic neurons [35]. Interestingly, at this later stage cell proliferation in the gill slits and dorsal cord was less pronounced, but a large number of EdU^+^ cells were found mid-intestinal in the trunk, potentially identifying a new region of posterior growth [68].

As *S. californicum* transitioned from a distinct larva through metamorphosis and into a juvenile, proliferative cells shifted in their distribution, restricting to specific regions in the juvenile body. Overall, our data suggests that the proliferation of the adult body plan begins at late larval stages prior to the start of the metamorphosis itself.

### RNAseq after irradiation reveals the genetic signature of proliferative cells in two distinct life history states

To further explore the molecular characteristics of proliferative cells in *S. californicum*, we exploited the sensitivity of proliferative cells to irradiation [69–73]. We hypothesized that the transcripts of irradiation-sensitive genes would be restricted to our EdU^+^, proliferative cell population. When we inspected the morphology of EdU^+^ proliferative cells with fluorescent in situ hybridization (FISH) to detect histone h2b messenger RNA, a known cell cycle gene, we found that EdU^+^ cells possess a narrow rim of cytoplasm of h2b mRNA surrounding their nucleus and these cells often display a cytoplasmic projection (Additional file 1: S1G, H). This morphology is reminiscent of the proliferative cells studied in other organisms, such as planarian neoblasts, which have been characterized as rounded mesenchymal cells with a high nuclear-to-cytoplasmic ratio that often extend a cytoplasmic projection [74, 75]. With these additional characterizations we next wanted to know if these proliferative cells might share any core genetic signatures with proliferative cells in other organisms. One hypothesis was that *S. californicum* would have a stem-cell-like population that expresses many of the classic multipotency or germline multipotency factors, such as *piwi, vasa,* and *nanos* [71, 76].

**Fig. 7:**
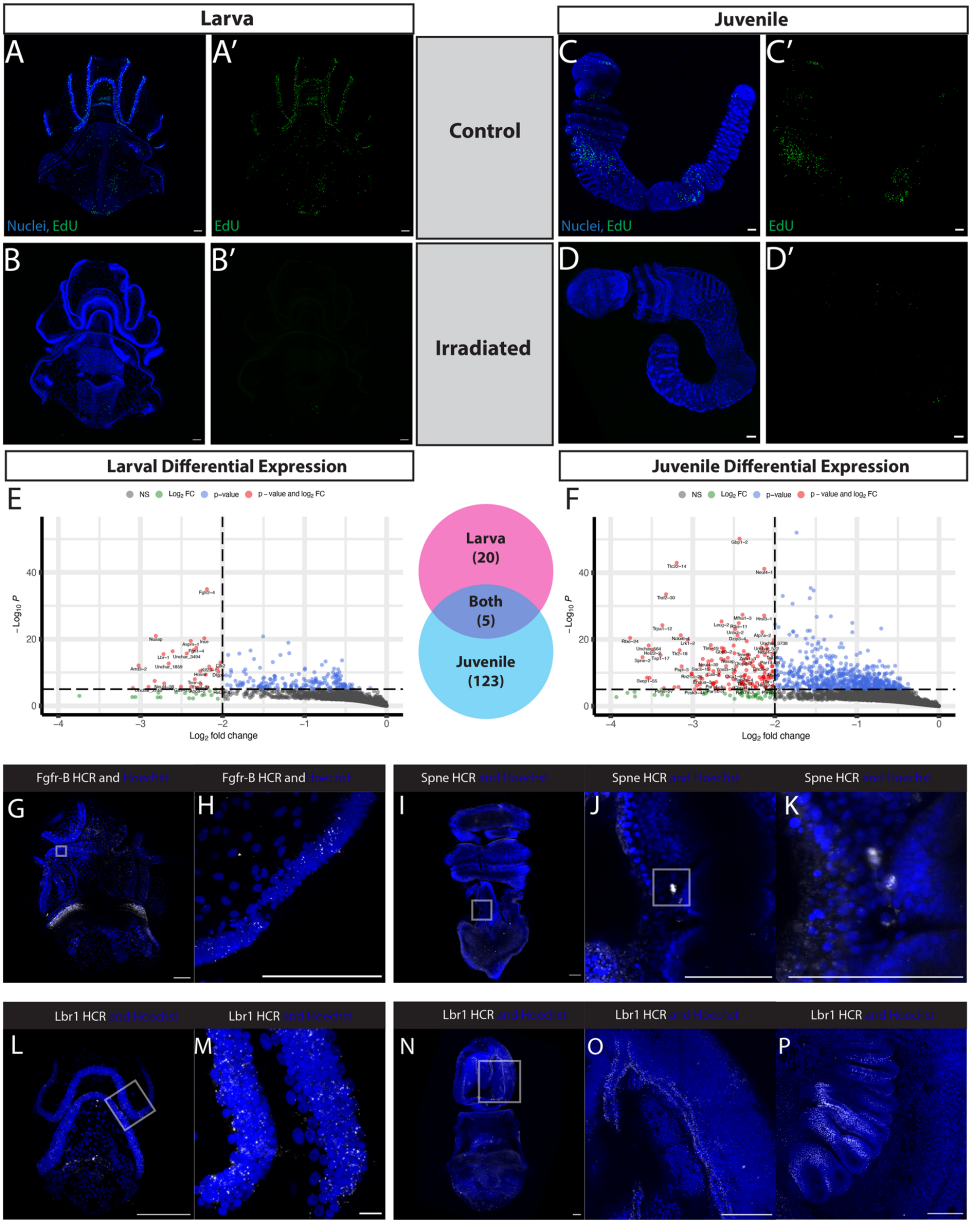
Genetic signature of irradiation sensitive EdU cells in both larvae and juveniles. All; blue = Hoechst, green = EdU; scale bar is 100um. **A** Control larva representing the normal EdU pattern at this stage, representative of 5/5 animals. **B** Experimental larva representing the EdU pattern at this stage after receiving 120 Gy of X-ray irradiation, representative of 5/5 animals. **C** Control juvenile representing the normal EdU pattern at this stage, representative of 4/4 animals. **D** Experimental juvenile representing the EdU pattern at this stage after receiving 200 Gy of X-ray irradiation, representative of 2/2 animals. **E** Volcano plot showing expression differences in control versus irradiated larva. n = 3 for each group. **F** Volcano plot showing expression differences in control versus irradiated juvenile. n = 3 for each group. **G** A larva with HCR probes for Fgfr-B. **H** Higher magnification of ciliary band with Fgfr-B transcripts. **I** A juvenile with HCR probes for Spindle-E. **J**, **K** Higher magnification of Spindle-E transcripts expressed between the ectoderm and the gills bars. **L** A larva with HCR probes for Lbr-1. **M** Inset of H with Lbr-1 transcripts distributed throughout the ciliary band. **N** A juvenile with HCR probes for Lbr-1. **O** Inset of I with Lbr-1 transcripts distributed throughout the lateral grooves. **P** Lbr-1 transcripts distributed in the gill bars

To do this, we treated larvae and juveniles with irradiation. Three days after treatment, animals looked morphologically the same as controls, but EdU incorporation was eliminated in both larvae and juveniles (Fig. 7A–D). We extracted total RNA from this same stage of three days post-irradiation from 5 pooled individuals in three independent biological replicates and made RNA sequencing libraries (Nugen-Tecan Genomics). RNAseq analysis of irradiated versus non-irradiated identified 20 genes in larvae and 123 genes in juveniles showing significant differential expression (log2 fold change ≥ − 2) and p-adjusted value ≤ 10–6 juveniles (Fig. 7E), with 5 genes that were downregulated at both stages.

In the larval stage, twenty candidate genes were specific, including *fgfr-B* (fibroblast growth factor receptor B) and a number of genes involved in cell division, such as *ince (*inner centromere protein) [77] *aspm-1* (abnormal spindle microtubule assembly*)* [78–80], and *dlgp5* (disks large-associated protein 5*)* [81, 82] (Additional file 2: S2A). In juveniles, 123 genes showed significant differential expression including a potential germline marker *spne-2 (*spindle-E), genes involved in proliferation, such as *anln* (anilin), and genes related to a potential immune response *traf2* (TNF receptor-associated factor 2), *tlr2* (Toll-like receptor 2), and *tlr6* (Toll-like receptor 6) (Additional file 2: S2B).

Finally, five genes with differential expression were shared between larva and juvenile: *lbr-1* (Lamin B Receptor), *nusap* (Nucleolar And Spindle Associated Protein 1), *tenr-5* (Tenascin-R), *tlr6-1* (Toll-like receptor 6), and *unchar_4293* (an uncharacterized gene). *nusap* plays a role in spindle microtubule organization and also has been implicated in WNT signaling and metastasis [83]*,* and *tenr-5* (Tenascin-R), which belongs to a group of extracellular matrix proteins, tenascins, which are important in vertebrates stem cell niches for tissue formation, cell adhesion modulation, and the regulation of proliferation and differentiation [84].

Among the larval irradiation-sensitive transcripts, *fgfr-B* was most notable. FGF receptors in vertebrates are known to regulate cell proliferation, differentiation, and play a key role in pluripotent stem cells [85]. The two hemichordate FGF receptors Fgfr-A and Fgfr-B arose from a hemichordate-specific duplication [86] and in the direct developing hemichordate *Saccoglossus kowalevskii, fgfr-B* is expressed in the endomesoderm at early gastrula stage and also in the ectoderm beginning at late gastrula into later stages [87]. In *S. californicum*, we examined the distribution of *fgfr-B* mRNA and found expression throughout regions, where we also had previously observed EdU^+^ cells, particularly in the ciliary bands (Fig. 7G, H).

In the juvenile transcriptomes, the differential expression of *spne-2 (*spindle-E) was most notable. In *Drosophila melanogaster* spindle-E is involved in the generation of germ cell piwi-interacting-RNAs (piRNAs) and the DExD-box helicase domain of s*pindle-E* is required for silencing of transposable elements in the germline [88, 89]*.* In *S. californicum, spindle-E* was specifically expressed in mesenchymal cells around the posterior of the gills bars (Fig. 7I, K), which is consistent with Vasa expression in *P. flava* [90]. Given that *spindle-E* was expressed in a similar region and has been implicated in germline regulation, we hypothesize that *spindle-E* could potentially be a marker of proliferative germline cells in hemichordates.

The lamin B receptor gene, *lbr-1*, which plays an important role in tethering chromatin, was differentially expressed in the larval and juvenile stages. This suggests it may be a universal marker of proliferative cells in *S. californicum*. There are two types of chromatin attachment to lamina, one type is executed by the lamin B receptor in embryonic and non-differentiated cells, and the other by specific lamin a/c binding proteins in differentiated cells [91]. Previous work in ascidians and echinoderms has identified *lbr-1* orthologs and suggested that this gene may be unique to deuterostomes [92]. We examined the expression of *lbr-1* in larvae and found it localized in the ciliary bands (Fig. 7L, M), which we previously demonstrated were regions of active cellular proliferation (Fig. 3A). Similarly, at the juvenile stage we found *lbr-1* expression in a similar territory, where we had observed the distribution of EdU^+^ cells (Figs. 5F, 6B, C), such as the lateral grooves in the proboscis (Fig. 7N, O) and in the gill bars (Fig. 7P). Our findings suggest that expression of *lbr-1* might serve as a useful marker of labeling proliferative cells across both life history states.

Finally, the classic multipotency or germline multipotency factors, such as *piwi, vasa,* and *nanos*, [71, 76] did not have significant differential expression (Additional file 2: S2C, D). Instead, what we recovered were genes related more to specific proliferative populations (*fgfr-B, spne-2,* and *lbr-1)* and thereby, revealed a possible heterogeneity among proliferative progenitor cells.

### Cell death remodels larval tissue at metamorphosis

After an investigation of cell proliferation throughout the life cycle and metamorphosis of *S. californicum,* we next tested if patterns of proliferation were correlated with patterns of cell death. One larval structure that is lost or extensively remodeled at metamorphosis is the circumoral ciliary band, also called the longitudinal ciliary band, a larval specific feeding structure [57, 61] that is not retained in the juvenile. We investigated the distribution of cell death with TUNEL (terminal deoxynucleotidyl transferase dUTP nick end labeling), which detects breaks in DNA as a proxy for cells undergoing apoptosis [93]. The TUNEL assay labels all free 3′-hydoxyl termini meaning that TUNEL staining will detect apoptosis, programmed cell death, but also necrosis [94, 95]. We overcame previously limitations of TUNEL detection by taking advantage of Click-iT technology, which utilizes a modified dUTP with a small, bio-orthogonal alkyne moiety (EdUTP) and a copper catalyzed covalent click reaction between that alkyne and a picolyl azide dye [96, 97].

**Fig. 8:**
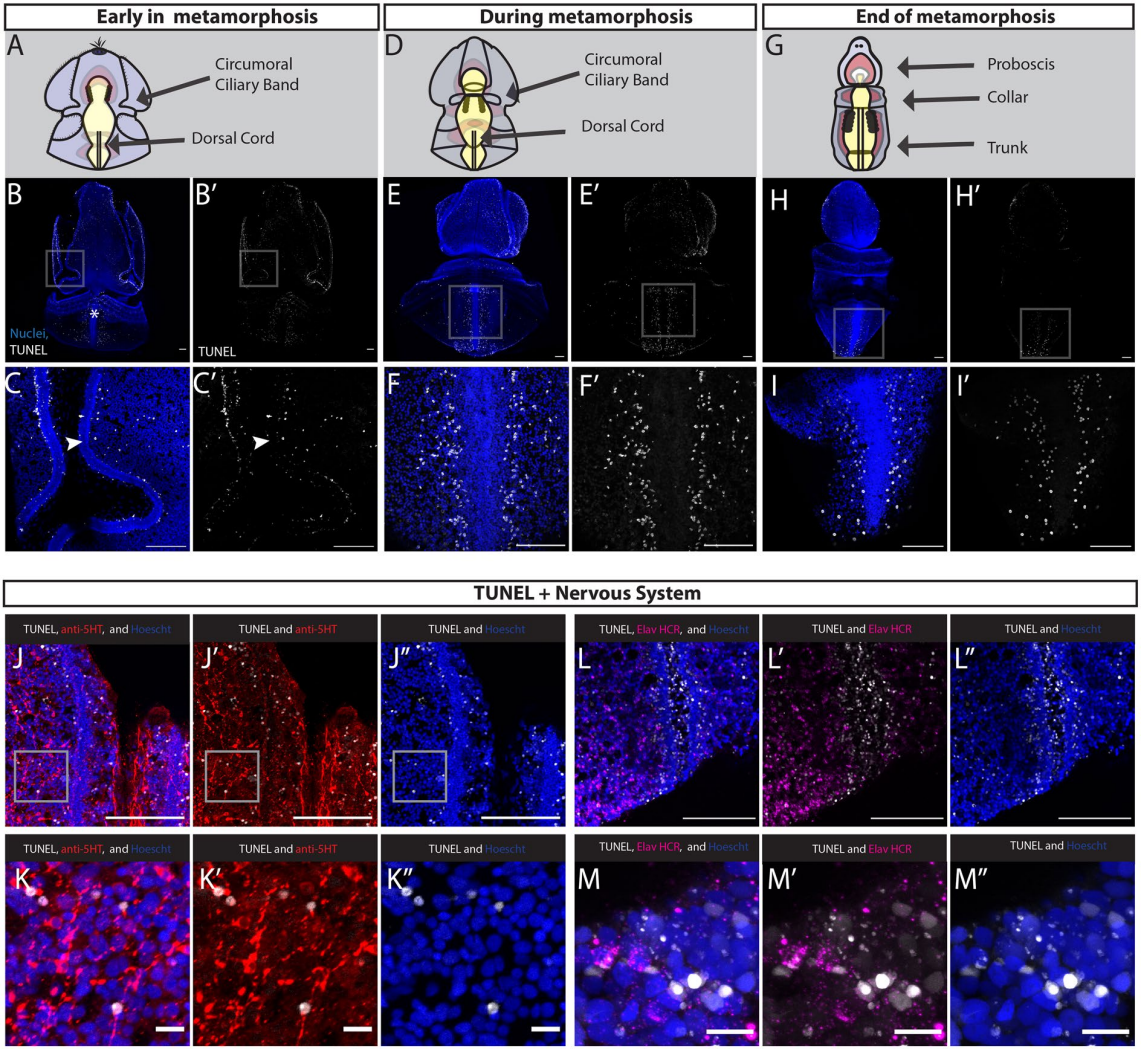
Cell death throughout the metamorphosis of *S. californicum.* All: anterior up; blue = Hoechst, grey = TUNEL; scale bar is 100um. Schematics of early in metamorphosis (**A**), during metamorphosis **(D**), and end of metamorphosis (**G**) with key structures labeled. **B** Start of metamorphosis with an increase in TUNEL^+^ cells. **C** Highlights regions of (**B**), specifically around lateral grooves. **E** Middle of metamorphosis. **F** Highlights regions of **E**, specifically around the dorsal cord. **H** End of metamorphosis. **I** Highlights regions of (**H**), specifically around the dorsal chord and collar. **J** Overlap of serotonin^+^ cells and TUNEL^+^ cells. **K** Inset of (**G**), highlighting TUNEL^+^ and serotonin^+^ positive cells. **L** Overlap of Elav^+^ cells and TUNEL^+^ cells. **M** Inset of (**L**), highlighting TUNEL^+^ and Elav^+^ cells

Throughout larval development and in late larva we detected few TUNEL^+^ cells, suggesting very limited cell death at larval stages (Additional file 3: S3A–C). However, once metamorphosis began, indicated by the thickening of the larval epithelium, there was a large increase in TUNEL^+^ cells (Fig. 8B). TUNEL^+^ cells were distributed broadly throughout the ectoderm, with most of them on either side of the developing dorsal cord, and in and around the circumoral ciliary band (Fig. 8C). The circumoral ciliary band was labelled with many TUNEL^+^ cells, supporting the morphological observation that this structure begins to break down at this stage. There were a small number of TUNEL^+^ at the anterior end of the protocoel (Additional file 3: S3D, E). TUNEL^+^ cells were also absent from the gut at this stage, which is consistent with the morphological observation that the gut is maintained throughout the transition from larvae to adult [35]. To confirm adequate penetration of the TUNEL labeling into the deeper tissue layers, we performed a positive control by artificially nicking the ends of DNA with DNAse-1 (Additional file 3: S3G, H).

At the mid-metamorphosis stage, TUNEL^+^ cells were distributed throughout the epidermis and continue to label the disintegrating ciliary bands that fuse with each other (Fig. 8E). At this stage we continued to detect very few TUNEL^+^ cells in the mesoderm and endoderm. The other region of the ectoderm, where the greatest number of TUNEL^+^ cells were found is directly lateral to the dorsal nerve cord (Fig. 8F), a region where we observed many proliferative cells at the same stage. These general patterns that we see on the dorsal side are broadly similar to what was observed on the ventral surface with TUNEL^+^ cells distributed in the ciliary bands and broadly in the ectoderm but excluded from the ventral nerve cord (Additional file 3: S3F). The presence of TUNEL^+^ cells in and around the ciliary bands was consistent with a previous observation that the circumoral ciliary band degenerates and the serotonergic nervous system in this region undergoes extensive reorganization during metamorphosis, as neurite bundles that are present in the ciliary grooves disappear as the ciliary bands fuse [35, 98, 99].

At the end of metamorphosis, there were fewer TUNEL^+^ cells detected. In the anterior, there were very few TUNEL^+^ cells in either the ectoderm or mesoderm (Fig. 8H). Those TUNEL^+^ cells that remain were scattered throughout the epidermis in the proboscis, but no longer in the lateral grooves, which from our EdU study we have observed becoming proliferative at this stage (Fig. 5F). In the posterior, the remaining TUNEL^+^ cells were detected on either side of the dorsal cord, most prominently in the posterior, where the larval epidermis had compacted (Fig. 8I).

To understand the interaction of cell death with the nervous system, we examined serotonin localization along with TUNEL early in metamorphosis and found there is colocalization of serotonergic^+^ cells with TUNEL^+^ cells (Fig. 8J, K). We also examined expression of *elv*, a pan-neuronal marker, with TUNEL (Fig. 8L, M) and found several colocalized *elv*^+^ and TUNEL^+^ cells at the edge of the epidermis. Our findings suggest that portions of the larval nervous system undergo cell death at metamorphosis and that the nervous system of the anterior ciliary bands may not be maintained in the juvenile body plan as previously proposed [66].

Overall, from our characterization of cell death, we found that an increase in TUNEL^+^ cells correlated with metamorphosis. We observed TUNEL^+^ cells broadly distributed in the epidermis and that their restriction over time, from anterior to posterior, correlated with the morphological observation of an anterior to posterior temporal progression of ectodermal thickening [35]**.** While we cannot rule out additional forms of tissue remodeling or histolysis, our findings suggest that cell death plays an important role in remodeling larval structures specifically in the anterior ciliary bands, which fused during metamorphosis.

## Discussion

While the development of an adult by transformation of a larva is very common in bilaterians, we understand very little about the details of how this process occurs at a cellular level, particularly given how the process of metamorphosis differs across groups with different life history strategies [11, 100–102]. This study in the hemichordate *S. californicum* focuses on characterizing the patterns of cellular proliferation and cell death during the two different life history stages and during metamorphosis. Unlike the model species *D. melanogaster*, where metamorphosis results in a major histolysis of larval tissues and the adult emerging from imaginal discs [103], morphological studies in *S. californicum* [35]*,* and indirect developing hemichordates broadly [36], suggest that metamorphosis occurs by remodeling of larval tissues and the transformation of larva into the adult. Our work illustrates the similarities and differences in patterns of proliferation and cell death across distinct life history states in *S. californicum* revealing how and when these unique body plans form.

### The larval body plan shaped by proliferation

For an organism with indirect development, rapid growth of the larva is essential. Eggs are small, yet juvenile size at metamorphosis is a good indicator of individual fitness, so larval growth before metamorphosis is critical [13, 104]. We observed in early larval development (Fig. 1B) that the tornaria larva is formed primarily through cell proliferation and limited amounts of cell death (Figs. 2, 3, Additional file 3: S3A–C). The patterns of proliferation we observed highlight regional differences of growth; EdU^+^ cells were distributed throughout the larval epidermis, the gut, and prominently in the ciliary bands (Figs. 2A, 3A). At this stage in larval development, we observed little cell death with the use of the TUNEL assay (Additional file 3: S3A). There were some TUNEL^+^ cells distributed throughout the larva, but it does not appear that these cells were concentrated to any structure or tissue. While cell death in bilaterian larval development has not been surveyed broadly across taxa, cell proliferation in larval development has been assessed in a number of marine larvae and the patterns we observe in *S. californicum* confirms and extends what has been found in other species [58].

### Origin of the adult body plan begins in the late larva

The morphological discontinuity between larvae and adults has been a consistent source of great curiosity for zoologists [30, 36, 105]. While metamorphosis is often thought of as the time when the adult animal emerges from the vestiges of larval anlage, cell proliferation may precede more overt morphological change at metamorphosis. In the late larva (Fig. 1D) of *S. californicum*, specific regions of the developing adult body are characterized by cell proliferation prior to the organism undergoing the transition from planktonic to benthic during metamorphosis (Fig. 4). At this stage, larvae are competent [9] to begin metamorphosis, but may remain as swimming tornaria for weeks or months. This is most obvious in the proliferation of the coeloms: the protocoel, mesocoel, and metacoel that give rise to the proboscis mesoderm, collar mesoderm, and trunk mesoderm, respectively (Fig. 4H–L). The formation of some of these morphological landmarks prior to metamorphosis has been described previously in *S. californicum* [35] and in enteropneusts broadly [36, 65, 99, 105] but our study clarifies that these structures originate via broad proliferation. Our work describing the patterns of proliferation illustrates the importance of proliferation in the origin of these tissues prior to the start of metamorphosis and supports long-standing hypotheses that the initiation of adult structures before metamorphosis is essential in preparing an organism for a major life history transition [2, 105, 106].

In indirect developing species, questions of adult origins revolve around when and where. The timing of adult formation in the sea urchin *Strongylocentrotus purpuratus* had been described as occurring through “set-aside cells” in which the juvenile grows from a small rudiment within the larval body from a population of sequestered cells [17]. Instead of a cellular identify definition of “set-aside cells”, we prefer a broader concept of heterochrony [107, 108] or “deferred development”, which focuses on timing of specification and terminal differentiation of some cell populations relative to others [109]. The paradigm of heterochrony or “deferred development” frames the adult development of S. *californicum* as more similar to the delayed life history shift of marine annelids than that of sea urchins [110–112]. In *S. californicum*, we observe this deferral of the adult in the late larva, where proliferation of structures of the adult body plan occurs *prior* to the start of metamorphosis (Fig. 4).

### Metamorphosis integrates cell proliferation and cell death

We found that cell death correlated with the onset of metamorphosis, and regionalized cell proliferation that began during late larval development continues into the adult. Cell death was detected in regions, where larval specific structures were remodeled (Fig. 8B, C) and likely is important in shaping the morphogenesis of emerging adult structures, such as the dorsal cord of the forming adult nervous system (Fig. 8E, F, H, I). Clearly the onset of adult morphogenesis, and the initiation of overt metamorphosis, results in a major shift in the patterns of proliferation and cell death.

Cell death within larval-specific structures has long been implicated in studies of metamorphosis, indeed one of the first recorded observation of apoptosis was in the metamorphosis of the toad, *Alytes obstetricans*, in which it was noted that cells of the notochord disappear and are replaced by cells of the vertebrae [113]. Since then, there have been important findings about the role of cell death during anuran metamorphosis that have extended the importance of timing in this process [114–116]. The mechanism of metamorphosis in insects such as butterflies and fruit flies have also provided important comparative perspectives into the role of programmed cell death as a key event in this process [117, 118]. Finally, cell death is implicated in the metamorphosis of marine invertebrates as they transition from planktonic larvae to benthic juveniles [119], in particular in the remodeling of the larval nervous system in gastropods [120, 121]. In *S. californicum* we detected TUNEL^+^ cells at the start of the morphological metamorphosis, most obviously around the anterior ciliary bands, which were involved in larval feeding, and fuse during metamorphosis (Fig. 8B). This is similar to what has been observed in sea urchins, where apoptotic cells were detected in the arms and ciliary bands of competent larvae [122–124]. Similar to what has been observed in the enteropneust *P. flava* [66]*,* in *S. californicum* the serotonergic neurons associated with ciliary bands are lost at metamorphosis and we observed TUNEL and serotonin double-positive cells suggesting cell death is one way these systems may change at metamorphosis (Fig. 8J, K).

While cell death plays an important role in removing larval-specific structures at metamorphosis, it may also be involved in sculpting larval tissue during metamorphosis, reminiscent of digit development in vertebrates [125], and the formation of leg joints and head segments in *D. melanogaster* [126]. One defining feature of metamorphosis in *S. californicum* is an overall decrease in size that occurs from anterior to posterior. Our finding that cell death proceeds from the anterior to posterior in the dorsal epidermis (Fig. 8E, F) suggest this might be an important process in integrating and removing larval tissue, similar to the apoptosis observed in the mouse paw or fly larva [127]. However, a more complete picture of the type of cell death, apoptosis or necrosis, or programmed cell removal [128] would need to be tested more rigorously with more sophisticated functional approaches. Overall, while we find that cell death occurs both in larval-specific structures but also broadly in larval tissue during metamorphosis, there are also regions with more limited cell death, such as the anterior ectoderm, collar ectoderm, and tripartite gut, leading to the possibility that not all larval cells die and may instead incorporated into the adult body plan. Our characterization of cell death in *S. californicum* supports classical morphological descriptions that enteropneusts do not have a “catastrophic metamorphosis” [129], and is now confirmed at a cellular level.

### Proliferative patterns of growth differ in the adult body plan, as do the markers of these proliferative populations

Juvenile *S. californicum* (Fig. 1J) have distinct patterns of growth—proliferation continues to be enriched in structures that were not functionally part of the larva. The dorsal and ventral cords, gill bars, proboscis ectoderm and lateral groove are all clear examples of regional proliferation of adult structures (Fig. 6B, Additional file 1: S1F). We also looked later in juvenile development and found that proliferation was most striking in the trunk region of the animal that continues to grow (Fig. 6F). This pattern of post-metamorphic growth is reminiscent of the posterior axis elongation by an extended period of posterior growth described in the direct-developing hemichordate *S. kowalevskii* [68].

Given that patterns of cell proliferation differed between larvae and juveniles, we wanted to test whether the genetic signature of proliferative cells was similar or different between the life history states, and if there were specific populations of pluripotent stem cells or broad populations of proliferative progenitors. In organisms such as colonial ascidians [130], acoels [131], flatworms [132, 133], cnidarians [134, 135] and sponges [136], adult stem cells retain the potential to produce both the germline and several somatic cell types, and suggest that there may be an ancestral animal stem cell [137]. We did not recover a clear pluripotent stem cell population, in contrast to organisms with clear neoblast populations, such as platyhelminthes and acoels [71]. Genes associated with multipotency or germline multipotency also did not have significant differential expression (Additional file 4: S4C, S4D). Instead, we found differential expression of genes, such as *lbr-1* in both larvae and juveniles, pointing towards the importance of chromatin state independent of the type of proliferative cell. It was previously suggested that the patterns of *lbr* and *lamA/C* expression in a number of different mammalian cell types may be indicative of peripheral heterochromatin tethers regulating differentiation and perhaps this is a larger uniting trend across deuterostomes [91].

When we looked at the differential expression in irradiated vs. non-irradiated larvae, we found a relatively limited number of significantly differentially expressed genes, such as those involved in cell division (such as *ince, asmp-1, dlgp5*) and most interestingly *fgfr-B* (fibroblast growth factor receptor B). A similar approach in the parasite *Schistosoma mansoni* found FGF receptors were downregulated in response to irradiation, and further showed that the inhibition of FGF signaling with RNAi resulted in reduced EdU incorporation and down regulation of cell-cycle-associated transcripts [138]. In juvenile animals, while we did recover the expression of what may be a germline specific marker in *spindle-E*, there were also a number of transcripts from our irradiation experiment that suggest a potential immune response, such as *traf2*, *tlr2* and *tlr6*. We conclude that the differential expression of these genes is likely related less to the depletion of irradiation-sensitive transcripts and more likely an immune reaction in response to the irradiation.

While additional work will need to be done to determine if markers, such as *fgr-B* and *spindle-E* are lineage and life history-specific proliferation markers, our results certainly support the hypothesis that formation of larval and juvenile structures draws on distinct sets of proliferative populations. The strong focus on species with direct development may miss some interesting regulatory features of distinct proliferative cell populations related to the development of complex life cycles. For a fuller understanding of developmental diversity and how it has shaped animal body plans, we need both a broader phylogenetic sampling but also greater representation of complex developmental strategies.

### Integration of larval and adult body plans

Our study of the balance and timing between cell proliferation and cell death illustrates that adult morphological elements proliferate prior to the start of metamorphosis, and that the onset of metamorphosis correlates with the onset of cell death. However, this study has also raised more questions, particularly the mysterious fate of most larval cells that are seemingly maintained through the threshold of metamorphosis. The most provocative and exciting possibility is the potential of larval cells taking on new identities in the adult body plan. In sea urchins, which were the key example of catastrophic metamorphosis with set-aside cells, the organization of the larval epithelium is preserved as regionalized apoptosis occurs in the larval arms that are resorbed [124]. Morphological studies of other echinoderms such as sea cucumbers and cidaroids suggest that much of their larval epidermis is maintained into the juvenile stage and is not lost at metamorphosis [139, 140]. Even in examples, such as nemerteans, which are described as “maximally-indirect developers'' [141], we now know that the cells that create the imaginal discs also contribute to the larval body [142]. Even in *D. melanogaster* with its specialized imaginal disc cells, differentiated larval tracheal cells become proliferative and form the adult trachea and also adult-specific air sacs [143, 144]. In the tobacco hornworm, *Manduca sexta,* differentiated cells of the larval legs contribute to the adult legs [145]. Perhaps this linkage between larval and adult cells is quite common. While genetic tools would need to be developed to study this in detail, studies of transformational metamorphosis have the potential test whether transdifferentiation of cell types occurs as part of normal ontological development in organisms with complex life histories. If this is indeed true, we are left with a tantalizing question, when many larval cells remain, how do they take on the appropriate function in the adult?

Almost all our understanding of adult development comes from direct developers, where the adult body plan emerges from the embryo. Despite the prevalence and phylogenetic breadth of species that represent indirect development, we understand very little about how adult development occurs by transformation. Clearly a greater focus is needed on the range of development strategies that characterize metamorphosis in metazoans. Only through this broader sampling of life history strategies can we hope for a more comprehensive understanding of the developmental mechanisms responsible for adult body plan formation.

## Conclusions

Our study describes cell proliferation and cell death through the development of the indirect developing *S. californicum*. This species has distinct larval and adult body plans and like other indirect-developing hemichordates [31, 33, 98] a metamorphosis that is more transformational than catastrophic. Our data represent a cellular investigation into the common, yet understudied, bilaterian developmental strategy of formation of adult body plan by transformation of a larval body plan. Despite the prevalence of this life history strategy, we have little developmental insights into how this process occurs. Our study uncovered that the broad proliferation of adult body plan components starts prior to any overt metamorphosis. Although cell death was a prominent feature of metamorphosis and adult body plan development, it is unlikely that the entire cell complement of the adult can be explained by larval cell death and proliferation of a distinct adult stem cell population. Future studies will be needed to clarify the fate of larval cells through metamorphosis. Altogether, our study establishes a cellular characterization of the formation of larval and adult body plans and transitions through metamorphosis in *S. californicum,* an important species for understanding this transformational process through the lens of cell, developmental, and evolutionary biology.

## Methods

### Collecting, spawning, and larval rearing

Adult *Schizocardium californicum* were collected in Morro Bay State Park, California, in a mudflat located at 35°20′56.7″N 120°50′35.6″W with appropriate state permitting. Animals were spawned as described in [35] with individual females transferred in bowls of filtered seawater and placed in an illuminated incubator at 24–26 °C. Once hatched, larvae were transferred to 1 gallon glass jars with continuous stirring and fed larvae with a 1:1 mix of *Dunaliella tertiolecta* and *Rhodomonas lens*. Every two to four days, containers are washed, water was replaced with clean filtered seawater, and fresh algae was added. To grow larger numbers of animals, some larvae were placed on a continuous flow-through system by being transferred into diffusion tubes [146]. Once animals began metamorphosis, they were transferred into glass bowls with terrarium sand.

### EdU labeling

Labeling and detection of proliferating cells were performed using the Click-it Plus EdU 488 Imaging Kit (Life Technologies), with the following modifications. Larva and juvenile worms were cultured in FSW supplemented with 10 μM EdU diluted from a 10 mM stock in DMSO. Unless otherwise noted, animals were pulsed for 30 min with EdU then fixed with 3.7% paraformaldehyde in MOPS fix buffer (0.1 M MOPS, 0.5 M NaCl, 2 mM EGTA, 1 mM MgCl2, 1× PBS) for 1 h at room temperature (RT). For detection of EdU incorporation, labeled embryos were transferred to a solution of PBS and the detection was performed following the manufacturer’s protocol with an increased permeabilization time in 0.5% Triton® X-100 in PBS of 40 min and an increased detection time of 45 min.

### TUNEL detection

Detection of apoptotic cells were performed using the Click-iT Plus TUNEL Assay for in situ apoptosis detection with Alexa FluorTM dyes, with the following modifications. Animals were fixed with the standard protocol (3.7% paraformaldehyde in MOPS fix buffer), washed twice in 1 × PBS and permeabilized with proteinase-K for 15 min at room temperature. TdT reaction mixture was incubated for 60 min at 37 °C and Click-iT Plus reaction was carried out for 30 min at 37 °C.

### Antibody labeling

Fixation and antibody labeling was performed as described previously [35]. To visualize proliferative cells, we used a rabbit polyclonal anti-histone H3 (phospho S10) (Abcam ab5176) diluted 1:200 in blocking solution. To visualize cilia, we used a mouse monoclonal anti-acetylated tubulin antibody (Sigma T7451) diluted 1:400 in blocking solution. To visualize the serotonergic nervous system, we used a rabbit anti-serotonin antibody (Sigma S5545) diluted 1:300 in blocking solution. Secondary antibodies (ThermoFisher, Alexa Fluor) were added at 1:1000 dilution to the blocking solution.

### Imaging

Nuclei were stained with Hoechst 33,342 (1:1000) in PBS and mounted in PBS using coverslips elevated with clay feet. For juvenile worms, samples were transferred into 50% glycerol for 30–60 min, then 70% glycerol for imaging. Samples were imaged on a Zeiss LSM 700 with 10X, 20X and 40× objectives. For samples larger than the field of view, maximal intensity projections from several stacks were stitched together (Fiji).

### Irradiation and transcriptional profiling

Larva and juvenile worms were exposed to 120 and 200 Gy of X-ray irradiation on a CellRad Faxitron source. Animals were cultured in FSW after irradiation for 3 days and purified total RNA was prepared from pools of 5 animals using Qiagen RNeasy. Three independent biological replicates were performed for both control and irradiated experimental groups. Individually tagged libraries for RNA-seq were prepared (Nugen-Tecan Genomics Universal mRNA-seq Kit), pooled in a single lane, and 75-bp pair-reads were generated using an Illumina HiSeq2000 at the Chan-Zuckerberg Biohub. The resulting reads were mapped to the annotated *S. californicum* genome (v2.0) using CLC Genomics Workbench (CLC Bio) and differential expression was conducted with DeSeq2. *apeglm* was used for log fold change shrinkage [147] and *v*st (variance stabilizing transformation) was used for visualization [148].

### In-situ hybridization

Samples were relaxed using 3.5% MgCl2 prior to fixation and fixed in 3.7% formaldehyde in MOPS fix buffer for 1 h at room temperature (RT), washed in fix buffer, dehydrated in 100% ethanol and stored at − 20 °C. Genes were amplified from stage specific cDNA with random hexamers and cloned into pGEM-T Easy (Promega). Digoxygenin labeled antisense probes were synthesized using SP6 or T7 RNA polymerase (Promega). In situ hybridization was performed a combination of what has been described previously [52, 149]. RNA probes were diluted to 0.1–1 ng/ml and hybridized overnight at 60 °C and visualized using an Anti-DIG AP antibody and TSA-Cy3.

### In situ HCR version 3.0.

Complementary DNA sequences specific to genes of interest were submitted to the in situ probe generator from the Ozpolat Lab [150]. Gene orthology was determined by collecting sequences of interest from related species and then building gene trees. Sequences were aligned with MUSCLE [151] and trees were calculated with Bayesian inference trees using MrBayes version 3.1.2 [152] in 1,000,000 generations with sampling of trees every 100 generations and a burn-in period of 25% (Additional file 4: S4). The sequences generated by the software were used to order DNA oligo pools (50 μmol DNA oPools Oligo Pool) from Integrated DNA Technologies, resuspended to 1 μmol/μl in 50 mM Tris buffer, pH 7.5. HCR amplifiers with fluorophores B1-Alexa Fluor-546, B2-Alexa Fluor-488, and B3-Alexa Fluor-647 were ordered from Molecular Instruments, Inc. The HCR was performed based on Choi et al., 2018 and the Hybridization Chain Reaction (HCR) In Situ Protocol from the Patel Lab [153, 154]. For experiments that involved HCR and TUNEL labeling, HCR was conducted first, and then additional labeling was performed after.

## Supplementary Information

**Additional file 1: S1.** Additional characterization of proliferative cells in *S. californicum*. A) Bar chart of number of Hoesch + in the ciliary bands vs. non ciliary bands, error bars are ± 1 SD (66% Confidence interval). B) Bar chart of EdU + cells in the ciliary bands vs. non ciliary bands, error bars are ± 1 SD (66% Confidence interval). C–F) All: anterior up; scale bar is 100um. blue = Hoechst, green = EdU. C) Ventral view of EdU distribution early in metamorphosis. D) Ventral view of EdU distribution in the middle of metamorphosis. E) Lateral view of EdU distribution at the end of metamorphosis. F) Ventral view of EdU distribution in juveniles. G–H) Expression of h2b mRNA and EdU positive cells in the juvenile proboscis blue = Hoechst, yellow = h2b mRNA, green = EdU.

**Additional file 2: S2. **Differential expression of larval and juvenile transcriptomes, irradiated versus non-irradiated. A) Differential expression of larval transcriptomes with baseMean, log2FoldChange, lfcSE, pvalue, padj. B) Differential expression of juvenile transcriptomes with baseMean, log2FoldChange, lfcSE, pvalue, padj. C) Differential expression of *piwi, vasa,* and *nanos* in larvae with baseMean, log2FoldChange, lfcSE, pvalue, padj. D) Differential expression of *piwi, vasa,* and *nanos* in juveniles with baseMean, log2FoldChange, lfcSE, pvalue, padj.

**Additional file 3: S3.** Additional characterization of TUNEL during larval development and metamorphosis. All: blue = Hoechst, grey = TUNEL, scale bar is 100um. A) lateral of view of mid tornaria body plan. B) Late larva with very few TUNEL + cells. C, Highlights regions of B) a few TUNEL + cells. C). D) Early in metamorphosis from Fig. 7C with an increase in TUNEL + cells. E) TUNEL + cells found in the mesodermal protocoel. F) Ventral view during the middle of metamorphosis. G) Positive control of TUNEL labeling by artificially nicking the ends of DNA with DNAse-1 metamorphosis H) Inset of positive control with TUNEL detected in deeper tissue layers

**Additional file 4: S4.** Gene Trees of HCR candidate Genes. Gene trees for A) Lbr-1 B) Fgfr C) Spne-2

## Data Availability

Sequencing raw reads and processed counts matrices associated with this study are available in the NCBI Gene Expression Omnibus (GEO), https://www.ncbi.nlm.nih.gov/geo/query/acc.cgi?acc=GSE196326.
