# Supplementary figures and images for: Comparisons of cell proliferation and cell death from tornaria larva to juvenile worm in the hemichordate Schizocardium californicum
Source: EvoDevo. 2022 Jun 6;13:13. doi: 10.1186/s13227-022-00198-1 (PMC9169294; doi:10.1186/s13227-022-00198-1)

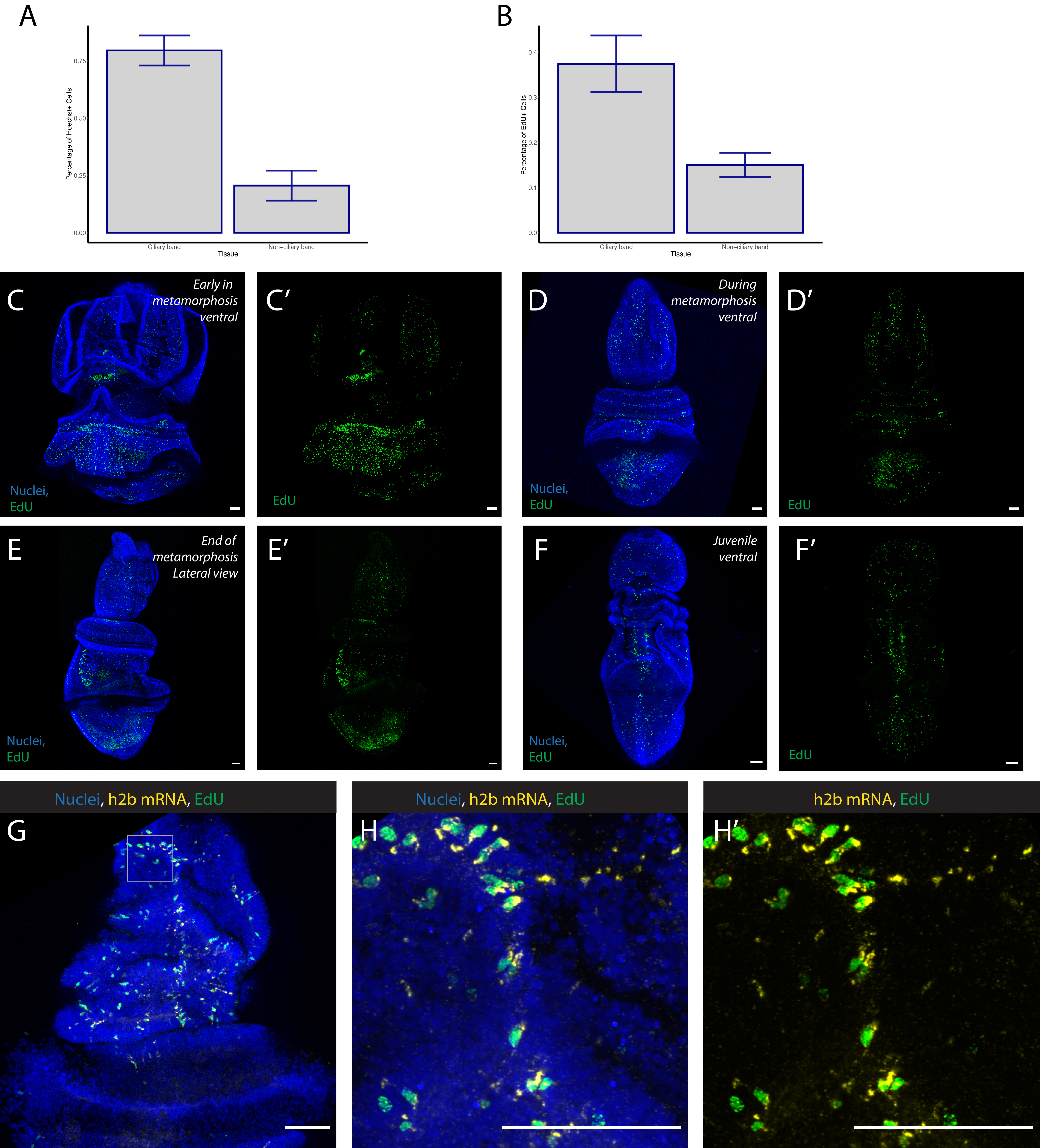

Supplement: Supplementary file 1 — Additional file 1: S1. Additional characterization of proliferative cells in S. californicum. A) Bar chart of number of Hoesch + in the ciliary bands vs. non ciliary bands, error bars are ± 1 SD (66% Confidence interval). B) Bar chart of EdU + cells in the ciliary bands vs. non ciliary bands, error bars are ± 1 SD (66% Confidence interval). C–F) All: anterior up; scale bar is 100um. blue = Hoechst, green = EdU. C) Ventral view of EdU distribution early in metamorphosis. D) Ventral view of EdU distribution in the middle of metamorphosis. E) Lateral view of EdU distribution at the end of metamorphosis. F) Ventral view of EdU distribution in juveniles. G–H) Expression of h2b mRNA and EdU positive cells in the juvenile proboscis blue = Hoechst, yellow = h2b mRNA, green = EdU. [file 13227_2022_198_MOESM1_ESM.jpg]

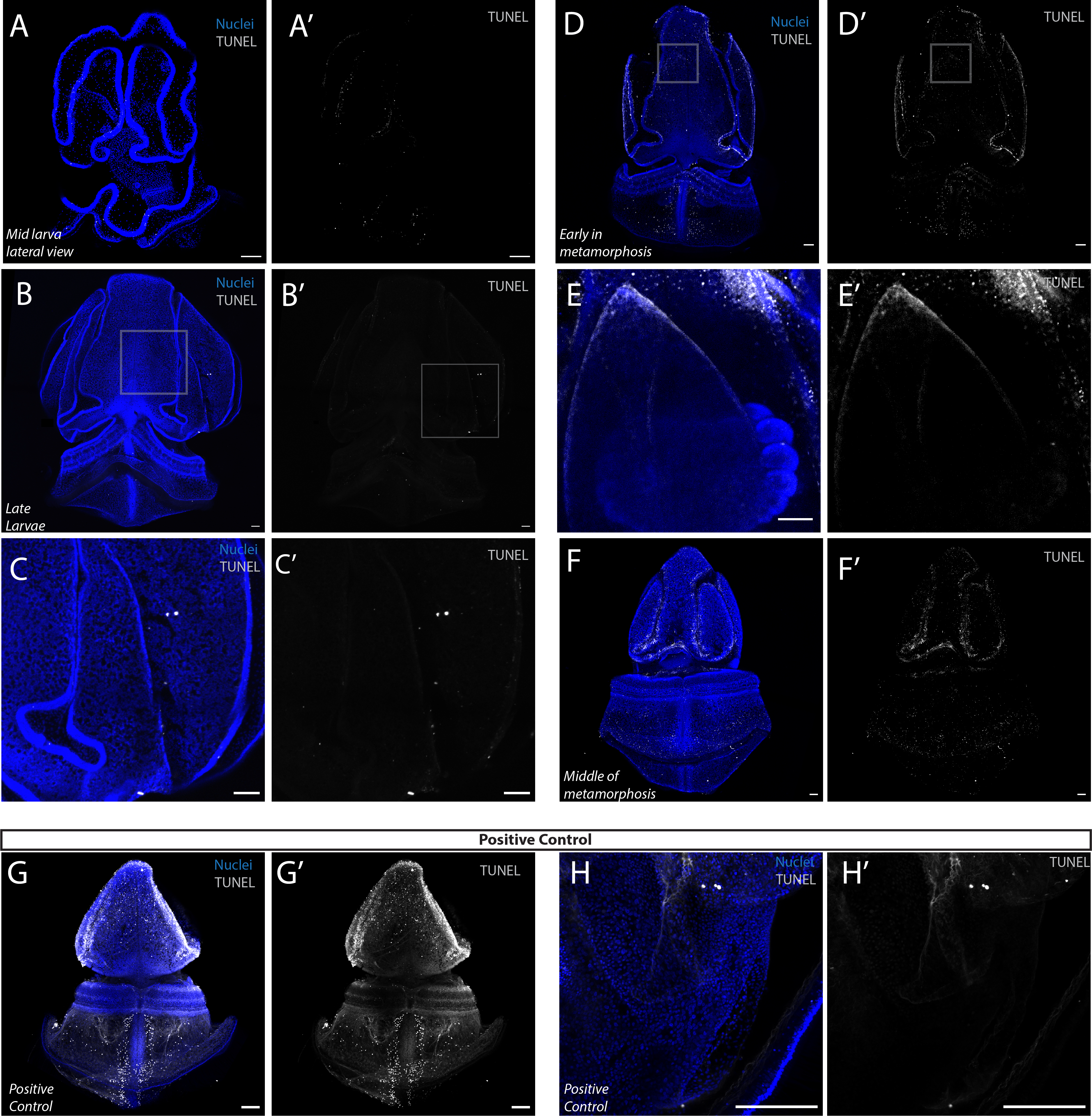

Supplement: Supplementary file 3 — Additional file 3: S3. Additional characterization of TUNEL during larval development and metamorphosis. All: blue = Hoechst, grey = TUNEL, scale bar is 100um. A) lateral of view of mid tornaria body plan. B) Late larva with very few TUNEL + cells. C, Highlights regions of B) a few TUNEL + cells. C). D) Early in metamorphosis from Fig. 7C with an increase in TUNEL + cells. E) TUNEL + cells found in the mesodermal protocoel. F) Ventral view during the middle of metamorphosis. G) Positive control of TUNEL labeling by artificially nicking the ends of DNA with DNAse-1 metamorphosis H) Inset of positive control with TUNEL detected in deeper tissue layers [file 13227_2022_198_MOESM3_ESM.jpg]

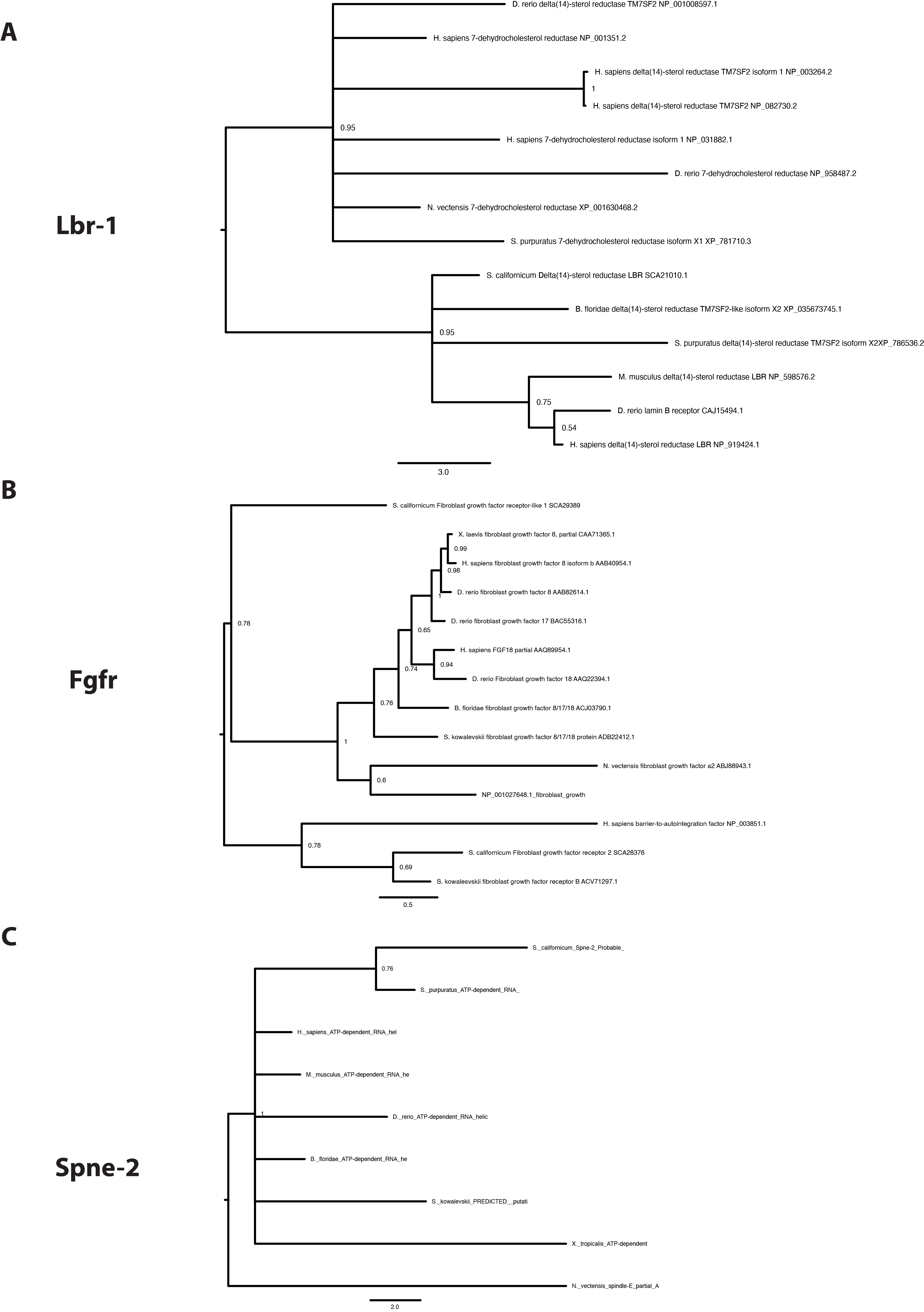

Supplement: Supplementary file 4 — Additional file 4: S4. Gene Trees of HCR candidate Genes. Gene trees for A) Lbr-1 B) Fgfr C) Spne-2 [file 13227_2022_198_MOESM4_ESM.jpg]
