# Supplementary material for: Comparisons of cell proliferation and cell death from tornaria larva to juvenile worm in the hemichordate Schizocardium californicum
Source: EvoDevo. 2022 Jun 6;13:13. doi: 10.1186/s13227-022-00198-1 (PMC9169294; doi:10.1186/s13227-022-00198-1)
Supplement: Supplementary file 2 — Additional file 2: S2. Differential expression of larval and juvenile transcriptomes, irradiated versus non-irradiated. A) Differential expression of larval transcriptomes with baseMean, log2FoldChange, lfcSE, pvalue, padj. B) Differential expression of juvenile transcriptomes with baseMean, log2FoldChange, lfcSE, pvalue, padj. C) Differential expression of piwi, vasa, and nanos in larvae with baseMean, log2FoldChange, lfcSE, pvalue, padj. D) Differential expression of piwi, vasa, and nanos in juveniles with baseMean, log2FoldChange, lfcSE, pvalue, padj. [file 13227_2022_198_MOESM2_ESM.pdf]

A. Larval Irradiation Candidates

	baseMean	log2FoldChange	lfcSE	pvalue	padj
**Fgfr2-4**	1630.175	-2.18984	0.179364	1.04E-35	3.34E-32
**Nusap**	89.84436	-2.81307	0.303536	1.09E-21	2.33E-18
**Ince**	119.9263	-2.22164	0.244389	5.53E-21	8.21E-18
**Aspm-1**	162.5113	-2.38698	0.268418	3.50E-20	4.83E-17
**Fgrl1-4**	91.02211	-2.31941	0.277801	3.58E-18	3.63E-15
**Mib1-3**	242.9705	-2.35456	0.288561	2.08E-17	1.74E-14
**Tlr6-1**	61.56051	-2.60842	0.323066	4.34E-17	3.35E-14
**Unchar_3494**	370.3807	-2.43616	0.310436	1.99E-16	1.37E-13
**Lbr-1**	77.78669	-2.71911	0.347314	3.07E-16	1.98E-13
**Ctk2**	91.29794	-2.02464	0.288236	1.07E-13	5.14E-11
**Unchar_1859**	33.12484	-2.65533	0.378874	2.02E-13	9.05E-11
**Arrd3-2**	36.35581	-3.02327	0.446694	8.14E-13	3.41E-10
**Kif23-1**	90.78675	-2.16175	0.32543	1.76E-12	7.21E-10
**Unchar_4293**	135.4392	-2.06022	0.317639	5.08E-12	1.92E-09
**Ki11a**	82.03937	-2.13209	0.336116	1.26E-11	4.26E-09
**Hmmr**	41.41535	-2.26999	0.367991	3.87E-11	1.19E-08
**Dlgp5**	84.32673	-2.04988	0.336565	5.70E-11	1.64E-08
**Garem-1**	50.29227	-2.20376	0.379007	4.43E-10	1.07E-07
**Tenr-5**	47.0752	-2.33731	0.442723	1.02E-08	1.76E-06
**Helz2-7**	116.1244	-2.82684	0.572553	3.50E-08	5.19E-06

B. Juvenile Irradiation Candidates

	baseMean	log2FoldChange	lfcSE	pvalue	padj
**Gbp1-2**	341.796	-2.4307	0.163921	6.35E-51	2.65E-47
**Ttc22-14**	145.5675	-3.19616	0.233735	1.10E-43	3.83E-40
**Neul4-1**	451.0323	-2.12873	0.159312	6.89E-42	2.05E-38
**Traf2-30**	1131.106	-3.3257	0.27781	2.92E-34	5.08E-31
**Mfha1-3**	552.3618	-2.39307	0.222611	3.92E-28	3.71E-25
**Hrsl3-1**	685.2498	-2.13015	0.199273	6.85E-28	6.20E-25
**Lecg-2**	203.9375	-2.64955	0.256702	3.95E-26	3.04E-23
**Rtbs-11**	100.8882	-2.43979	0.23934	1.28E-25	9.17E-23
**Trpa1-12**	223.4717	-3.37139	0.335112	5.49E-25	3.47E-22
**Un5cl-2**	1140.134	-2.48357	0.254042	8.08E-24	4.43E-21
**Atp7a-2**	1473.088	-2.15204	0.224653	6.02E-23	2.79E-20
**Nckx6-4**	135.4189	-3.14621	0.337107	5.62E-22	2.28E-19
**Dzip3-4**	105.7859	-2.45534	0.2627	6.03E-22	2.37E-19

**Rtxe-24**	341.8183	-3.76393	0.411762	3.63E-21	1.28E-18
**Lrk1-2**	208.5048	-3.06119	0.341051	1.44E-20	4.76E-18
**Unchar_3738**	530.598	-2.01175	0.223771	1.49E-20	4.85E-18
**Unchar_4293**	135.4392	-2.34311	0.262811	3.71E-20	1.17E-17
**Unchar_736**	239.0824	-2.02552	0.231088	1.18E-19	3.51E-17
**Tlr6-19**	75.49353	-2.78081	0.321575	4.99E-19	1.28E-16
**Unchar_522**	340.8634	-2.102	0.244208	5.14E-19	1.31E-16
**Unchar_564**	772.3732	-3.5374	0.414146	6.62E-19	1.64E-16
**Par14-36**	555.8046	-2.50209	0.293003	7.23E-19	1.77E-16
**Fcgbp-5**	97.62039	-2.1658	0.257802	2.99E-18	6.77E-16
**Par14-22**	499.0506	-2.64666	0.316978	3.70E-18	8.20E-16
**Gbp3-2**	51.52854	-2.62133	0.313873	6.06E-18	1.29E-15
**Neul4-22**	361.8735	-2.08839	0.251677	6.94E-18	1.46E-15
**Anln**	106.3931	-2.76097	0.32943	8.41E-18	1.72E-15
**Helz2-2**	471.173	-3.49158	0.425984	1.32E-17	2.62E-15
**Tlr2-18**	41.51185	-3.15919	0.38663	1.75E-17	3.45E-15
**Dtx3l-48**	690.1412	-2.1444	0.26339	2.23E-17	4.35E-15
**Tenr-5**	47.0752	-2.37845	0.293253	3.27E-17	6.11E-15
**Rn213-22**	182.4998	-2.62322	0.324544	4.26E-17	7.72E-15
**Anr55-3**	100.0866	-2.62807	0.325937	4.63E-17	8.32E-15
**Sacs-21**	286.0351	-2.49152	0.314536	1.37E-16	2.29E-14
**Toll-6**	295.0914	-2.03062	0.258807	3.10E-16	4.94E-14
**Znfx1-2**	920.7174	-2.33684	0.299294	3.34E-16	5.26E-14
**Ern1-3**	245.7098	-2.12359	0.272442	4.65E-16	7.22E-14
**Tsp1-17**	108.5286	-3.39356	0.436258	5.14E-16	7.94E-14
**Znfx1-3**	856.5053	-2.33028	0.301252	6.01E-16	9.13E-14
**Unchar_3199**	329.0729	-2.2087	0.288682	1.22E-15	1.78E-13
**Spne-2**	87.13935	-3.61232	0.477233	2.19E-15	3.05E-13
**Nusap**	89.84436	-2.57242	0.340659	3.51E-15	4.71E-13
**Tnf10-1**	199.3844	-2.3324	0.311223	4.19E-15	5.46E-13
**Gbp1-1**	57.22204	-2.65462	0.357632	6.41E-15	8.14E-13
**Ifih1-6**	239.7474	-2.87832	0.389052	7.22E-15	9.11E-13
**Par14-28**	3909.747	-2.05719	0.278852	9.81E-15	1.19E-12
**Oas3-2**	51.82069	-2.61656	0.356638	1.52E-14	1.82E-12
**Otop3-2**	154.7464	-2.38236	0.326566	1.83E-14	2.18E-12
**Helz2-1**	120.7123	-2.76886	0.379163	1.98E-14	2.31E-12
**Neul4-30**	31.62748	-2.80971	0.392604	3.92E-14	4.41E-12
**Dapk1-19**	100.0518	-2.27046	0.314664	4.09E-14	4.56E-12

**Unchar_738**	493.909	-2.30478	0.327647	1.10E-13	1.15E-11
**Mx1**	1858.205	-2.37222	0.33725	1.16E-13	1.21E-11
**Ddx58-4**	264.167	-2.34393	0.338489	2.61E-13	2.57E-11
**Sting-1**	400.6932	-2.18297	0.316439	3.05E-13	2.96E-11
**Dtx3l-2**	42.84706	-2.16878	0.320941	8.54E-13	7.46E-11
**Sacs-14**	353.1055	-2.80702	0.41642	8.66E-13	7.46E-11
**Sacs-15**	538.5808	-2.90482	0.432026	9.06E-13	7.74E-11
**Ymd3-16**	55.01933	-2.5941	0.385597	9.79E-13	8.26E-11
**Gbp3-1**	91.71746	-2.26544	0.3364	1.01E-12	8.45E-11
**Ncan-3**	123.4335	-2.02993	0.300826	1.04E-12	8.70E-11
**Arsb-63**	251.672	-2.03598	0.303042	1.12E-12	9.29E-11
**Papl-3**	30.60396	-3.1363	0.469825	1.19E-12	9.68E-11
**Sacs-9**	150.0414	-2.46959	0.369306	1.38E-12	1.10E-10
**Nada-3**	319.9379	-2.15271	0.32273	1.54E-12	1.22E-10
**M3k10**	306.63	-2.14718	0.328022	3.46E-12	2.56E-10
**Pcsk9-21**	137.2618	-2.80255	0.439654	8.86E-12	6.05E-10
**Ncam2-1**	220.0241	-2.34591	0.371796	1.58E-11	1.02E-09
**Ddx58-3**	180.8105	-2.12169	0.339126	2.17E-11	1.35E-09
**Unchar_922**	118.2275	-2.24251	0.357785	2.55E-11	1.57E-09
**Endub-3**	28.61787	-2.48477	0.399556	2.66E-11	1.62E-09
**Catl-2**	181.5082	-2.74919	0.444244	3.17E-11	1.91E-09
**Ifih1-5**	252.7791	-2.02858	0.327144	3.54E-11	2.11E-09
**Arsi-3**	259.1597	-2.02039	0.328784	4.57E-11	2.62E-09
**Unchar_3247**	51.38203	-2.6797	0.431023	5.60E-11	3.19E-09
**Clca1-6**	48.81313	-2.5027	0.418174	1.16E-10	6.14E-09
**Rn213-25**	21.3758	-3.01197	0.502621	1.52E-10	7.68E-09
**Unchar_2684**	171.8979	-2.12437	0.358005	1.87E-10	9.16E-09
**Trpa1-13**	37.40464	-3.0141	0.512492	2.68E-10	1.26E-08
**Par14-35**	31.14865	-2.05843	0.353369	3.26E-10	1.48E-08
**Paf15**	47.28724	-2.36046	0.406903	4.59E-10	2.02E-08
**Endua-1**	22.18769	-2.68102	0.464715	4.95E-10	2.16E-08
**Pgfra**	21.84278	-2.82684	0.494981	5.39E-10	2.32E-08
**Helz2-4**	87.13036	-2.37221	0.414769	6.80E-10	2.86E-08
**Unchar_2123**	400.4305	-2.08215	0.372844	1.37E-09	5.42E-08
**Rn213-23**	34.64558	-2.13958	0.381366	1.51E-09	5.88E-08
**Rab7a-4**	27.16251	-2.3785	0.427277	1.57E-09	6.09E-08
**Neul4-38**	49.2008	-2.57817	0.464363	1.76E-09	6.74E-08
**Unchar_1989**	44.5272	-2.14167	0.383324	1.77E-09	6.75E-08

**Sacs-10**	66.49401	-2.18558	0.395297	2.04E-09	7.60E-08
**Herc2-3**	297.6247	-2.02755	0.368037	2.37E-09	8.74E-08
**Mb213-5**	423.577	-2.13253	0.389559	2.55E-09	9.30E-08
**Cfdp2-64**	403.5795	-2.05362	0.377091	2.74E-09	9.97E-08
**Tlr2-16**	33.61337	-2.3182	0.421844	2.88E-09	1.04E-07
**Svep1-55**	39.12176	-3.55841	0.648675	3.07E-09	1.10E-07
**Lyam3-8**	20.61618	-3.52776	0.637637	3.39E-09	1.21E-07
**Pcsk9-28**	550.1161	-2.13902	0.394645	3.39E-09	1.21E-07
**Oas3-5**	30.92615	-2.90639	0.549108	4.34E-09	1.50E-07
**Lbr-1**	77.78669	-2.07277	0.38638	4.95E-09	1.67E-07
**Endua-3**	34.10096	-2.88468	0.538823	6.21E-09	2.04E-07
**Tlr2-23**	38.80898	-2.50797	0.474516	8.08E-09	2.57E-07
**Arsb-59**	32.80632	-2.16404	0.412927	9.18E-09	2.88E-07
**Pcsk9-9**	72.29667	-2.11567	0.406799	1.05E-08	3.23E-07
**Tlr2-17**	18.82426	-2.36485	0.459107	1.93E-08	5.58E-07
**Trpa1-15**	32.64893	-2.96569	0.578965	2.02E-08	5.82E-07
**Tlr6-23**	19.89627	-2.32801	0.464061	3.39E-08	9.13E-07
**Tlr6-16**	31.6292	-2.40429	0.486813	4.81E-08	1.24E-06
**Helz2-5**	112.955	-2.1012	0.427976	5.25E-08	1.34E-06
**Helz2-3**	775.969	-2.71311	0.559432	5.64E-08	1.42E-06
**Cfdp2-16**	21.57929	-2.41049	0.507699	9.22E-08	2.22E-06
**Susd2-4**	133.2866	-2.21769	0.466371	1.08E-07	2.56E-06
**Mb213-7**	70.49573	-2.75539	0.590883	1.22E-07	2.87E-06
**Par14-4**	104.2727	-2.73857	0.588614	1.41E-07	3.26E-06
**Unchar_2772**	125.4463	-2.15169	0.463596	1.85E-07	4.14E-06
**Helz2-8**	88.94362	-2.29908	0.497874	1.95E-07	4.33E-06
**Zo1-6**	39.69926	-2.39939	0.516723	2.00E-07	4.42E-06
**Anr52-3**	39.78255	-2.27627	0.493624	2.03E-07	4.47E-06
**Bgbp-2**	51.73825	-2.7608	0.600163	2.08E-07	4.56E-06
**Otop3-1**	85.54154	-2.75052	0.603507	2.45E-07	5.26E-06
**Otop2**	61.2041	-2.42398	0.534917	2.84E-07	5.98E-06
**Rn213-28**	805.1838	-2.56144	0.567377	2.91E-07	6.10E-06
**Cbpa4-1**	43.58815	-2.38222	0.528439	3.05E-07	6.33E-06
**Mb213-8**	45.59932	-2.91472	0.651967	3.14E-07	6.48E-06

C. Differential expression of “classic multipotency” genes in larva

	**baseMean**	**log2FoldChange**	**lfcSE**	**stat**	**pvalue**	**padj**

**Piwi-1**	93.74719	0.39905	0.371772	1.073373	0.283104	0.60958
**Nanos**	998.3336	-0.60208	0.280642	-2.14537	0.031924	0.187685
**Piwi-2**	130.5946	0.560572	0.277961	2.016727	0.043724	0.226244
**Vasa**	32.85435	-0.00313	0.434226	-0.00722	0.994243	1

D. Differential expression of "classic multipotency" genes in juveniles

	**baseMean**	**log2FoldChange**	**lfcSE**	**stat**	**pvalue**	**padj**
**Piwi-1**	93.74719	-0.3297	0.337528	-0.9768	0.32867	0.528603
**Nanos**	998.3336	-0.52775	0.28077	-1.87964	0.060158	0.165342
**Piwi-2**	130.5946	0.463554	0.288141	1.608776	0.107665	0.252103
**Vasa**	32.85435	-0.113	0.459135	-0.24611	0.805593	0.89698
